# Genome‐wide methylomic analyses identify prognostic epigenetic signature in lower grade glioma

**DOI:** 10.1111/jcmm.17101

**Published:** 2021-12-11

**Authors:** Wenna Guo, Shanshan Ma, Yanting Zhang, Hongtao Liu, Ya Li, Ji‐Tian Xu, Bo Yang, Fangxia Guan

**Affiliations:** ^1^ School of Life Sciences Zhengzhou University Zhengzhou China; ^2^ School of Basic Medical Sciences Zhengzhou University Zhengzhou China; ^3^ Department of Neurosurgery The First Affiliated Hospital of Zhengzhou University Zhengzhou China

**Keywords:** DNA methylation, immunotherapy, lower grade glioma, prognostic biomarker, regression analysis, risk stratification

## Abstract

Glioma is the most malignant and aggressive type of brain tumour with high heterogeneity and mortality. Although some clinicopathological factors have been identified as prognostic biomarkers, the individual variants and risk stratification in patients with lower grade glioma (LGG) have not been fully elucidated. The primary aim of this study was to identify an efficient DNA methylation combination biomarker for risk stratification and prognosis in LGG. We conducted a retrospective cohort study by analysing whole genome DNA methylation data of 646 patients with LGG from the TCGA and GEO database. Cox proportional hazard analysis was carried out to screen and construct biomarker model that predicted overall survival (OS). The Kaplan‐Meier survival curves and time‐dependent ROC were constructed to prove the efficiency of the signature. Then, another independent cohort was used to further validate the finding. A two‐CpG site DNA methylation signature was identified by multivariate Cox proportional hazard analysis. Further analysis indicated that the signature was an independent survival predictor from other clinical factors and exhibited higher predictive accuracy compared with known biomarkers. This signature was significantly correlated with immune‐checkpoint blockade, immunotherapy‐related signatures and ferroptosis regulator genes. The expression pattern and functional analysis showed that these two genes corresponding with two methylation sites contained in the model were correlated with immune infiltration level, and involved in MAPK and Rap1 signalling pathway. The signature may contribute to improve the risk stratification of patients and provide a more accurate assessment for precision medicine in the clinic.

## 1 INTRODUCTION

Glioma is one of the most common primary brain tumours in clinic, accounting for about 80% of all malignant brain tumors.^1^ According to WHO 2016 grading system, gliomas are categorized into lower grade and aggressive high‐grade gliomas, namely low‐grade gliomas (LGG) and glioblastoma (GBM).^2^ GBM is a deadly tumour with a median survival of only 15 months,^3^ and LGGs have a longer overall survival (OS), which varies from 1 to 15 years.^4^ Although LGGs have a relatively better prognosis, 70% of patients experience transformation to higher grade tumours within 10 years,^5^ indicating the importance of accurate assessment of outcomes in promoting individualized clinical management.^6^ The most recommended treatment for gliomas involves surgical removal followed by radiation and chemotherapy.^7^ In the clinic, LGG patients after surgery may be followed and determined the further radiotherapy or chemotherapy or not according to the comprehensive evaluation of clinical and molecular risk factors.^8^, ^9^ However, the effective and reliable biomarkers that could predict clinical outcomes and optimal therapeutic strategies are limited.

In recent years, some molecular markers and biological risk factors, such as MGMT (O6‐methylguanine DNA methyltransferase),^10^ IDH (isocitrate dehydrogenase),^11^ EGFR (epidermal growth factor receptor),^12^ PTEN (phosphatase and tensin homologue)^13^ and 1p/19q co‐deficiency^14^ have been discovered to be valuable for individualized therapeutic approaches and targeted therapies against GBM. However, there are few specific effective clinical indicators and therapeutic targets for LGGs. Moreover, studies have shown that patients may be similar in glioma grade but there are great differences in clinical prognosis and treatment response,^15^ so new effective molecular biomarkers are urgently needed to illuminate the mechanisms of LGG or to provide potential efficient therapeutic strategies.

RNA‐ or protein‐based information had been used to identify biomarkers for cancer, such as EGFR^12^ and PTEN.^13^ However, due to the highly dynamic nature of RNA and proteins in cancer biology and biological specimens, the information provided is sometime sunstable and had drawbacks in clinical application. Given that DNA is more stable than RNA or proteins over time, information based on DNA methylation may be more reliable. Changes of DNA methylation in cancer‐related gene promoter regions and CpG islands may lead to gene transcriptional silencing, and abnormal DNA methylation changes usually precede abnormal gene expression. Evaluation of DNA methylation can provide a more timely and accurate molecular information.^16^ Some DNA methylation inhibitors and cancer monotherapy have been approved by the Food and Drug Administration (FDA).^17^ Previous studies have shown that DNA methylation status is more reliable than gene expression in cancer diagnosis and prognosis, and DNA methylation detection using less tissues can identify the origin and predict the cancer prognosis efficiently and specifically independent of pathological examination.^18^, ^19^ Increasing studies have reported that the methylation of gene promoters is involved in key biological pathways in glioma.^20^ For example, promoter methylation of MGMT was found in approximately 40% of GBMs, and inactivation of the MGMT is associated with the clinical response to alkylating agents in glioma patients.^21^ Low MGMT levels were associated with modest improvements in response to temozolomide (TMZ) and survival.^22^ Yin et al^23^ reported a six‐CpG signature, which serve as a novel independent prognostic indicator for GBM. However, traditional biomarker discovery usually focused on known one or few genes, or differentially DNA methylation, lacking the systematic analysis method of genome‐wide DNA methylation, or lack of subsequent biomarker validation, which may suffer from false negatives. The single‐gene‐based epigenetic biomarkers such as MGMT had limited role in guiding clinical decision and failed to warrant a change in routine testing.^24^ Additionally since differentially DNA methylation may be completely independent of differences in survival, this may lead to poor performance of the model in predicting an unseen detection cohort.

In this study, the whole genome DNA methylation profiles of LGG samples from TCGA and GEO cohorts were analysed. Univariate and multivariate Cox regression analysis were adopted to screen the DNA methylation that associated with OS of patients with LGG. Sample‐splitting approach was used to segmented TCGA samples into discovery and validation cohorts. By using a robust likelihood‐based survival model and Cox regression analysis, we successfully identified a two‐CpG site DNA methylation signature that provides accurately survival risk stratification for patients with LGG and further evaluated the signature in the GEO cohort. In addition, the role of signature in tumour development and immunotherapy was further explored. These results indicate the potential of the DNA methylation signature in predicting the survival and immunotherapy of LGG patients and provide novel potential molecular therapeutic targets for LGGs.

## 2 MATERIALS AND METHODS

### 2.1 DNA methylation data and clinical characteristics of LGG populations

DNA methylation data (Infinium Human Methylation 450 BeadChip, level 3) and corresponding clinical information of LGG patients were obtained from TCGA database. For each DNA methylation site, methylation level is standardized as *β* value, which is the ratio of fluorescent signal that was measured by that of a methylated probe relative to the sum of the methylated and unmethylated probes, ranging from 0 (no methylation) to 1 (completely methylated). Here, patients with no exact survival information are filtered out. Finally, 514 cases with 485,577 DNA methylation sites from TCGA data set were included in this study. Two‐thirds of these cases were randomly selected as training cohort to identify and construct prognostic model, and the other one‐third were served as the validation cohort to evaluate the performance of the prognostic model. RNA sequencing (RNAseq) data were also downloaded from TCGA data set. The clinicopathological parameters related to this study were selected from the TCGA clinical data files for analyses, including age, gender, WHO grade, histologic subtype, IDH1 mutation status, radiation therapy status, family history of cancer and the latest clinical status. The distribution of patients is summarized in Table 1.

**TABLE 1:** The clinicopathological information of LGG patients from TCGA database

Characteristics	Groups	Total (*N* = 514)		Training cohort (*N* = 343)		Validation cohort (*N* = 171)	
		No	%	No	%	No	%
Gender	Male	285	55.45	188	54.81	97	56.73
	Female	229	44.55	155	45.19	74	43.27
Age at diagnosis	Median	41		58		58	
	Range	14–87		14–87		21–74	
	<41	253	49.22	187	54.52	66	38.60
	≥41	260	50.58	156	45.48	104	60.82
WHO grade	G 2	248	48.25	170	49.56	78	45.61
	G 3	265	51.56	172	50.15	93	54.39
	Unknown	1	0.19	1	0.29	0	0.00
Histologic subtype	Astrocytoma	194	37.74	128	37.32	66	38.60
	Oligodendroglioma	189	36.77	131	38.19	58	33.92
	Mixed glioma	131	25.49	85	24.78	46	26.90
IDH1 mutation	Yes	91	17.70	50	14.58	41	23.98
	No	34	6.61	18	5.25	16	9.36
	NA/Unknown	389	75.68	275	80.17	114	66.67
Radiation therapy	Yes	277	53.89	192	55.98	85	49.71
	NO	169	32.88	109	31.78	60	35.09
	Unknown	68	13.23	42	12.24	26	15.20
Family history of cancer	Yes	132	25.68	90	26.24	42	24.56
	NO	210	40.86	145	42.27	65	38.01
	NA/Unknown	172	33.46	108	31.49	64	37.43
Vital status	Alive	388	75.49	259	75.51	129	75.44
	Dead	126	24.51	84	24.49	42	24.56

In addition, 20 paired paraffin‐embedded human LGG tissues and the matched adjacent normal tissues were collected and sectioned in the First Affiliated Hospital of Zhengzhou University for immunohistochemistry. All procedures were conducted in accordance with guidelines provided by the Ethics Committees of First Affiliated Hospital of Zhengzhou University.

### 2.2 Statistical analyses

The statistical analyses were performed utilizing R software v3.6.1. Overall survival was defined as the duration from the date of diagnosis to the date of death or the last follow‐up. The univariate Cox regression analyses was performed to identify DNA methylation signatures that significantly related to the patients’ OS, and a cross‐validation approach was applied to analyse the robustness (*N* = 171, bootstrap = 100). DNA methylation sites with a two‐sided *p*‐value lower than 0.001 in more than 90 bootstraps were selected for further analysis. Then the multivariable Cox regression was used to further identify and construct the OS‐related prognostic model. We exhaustively selected two, three markers from the initial makers as covariates in cox regression and established models, and then AUC was used to measure and compare the model performance. We ranked the patients based on the risk scores that was calculated by using DNA methylation level and coefficient in multivariate Cox proportional hazards regression. The formula for calculating risk score is as following: Risk score = ∑ Coefficient of Cox proportional hazards regression of a DNA methylation × *β* value of the DNA methylation. Then, the prognostic risk score of each patient was calculated. According to the median risk score, LGGs patients were dichotomized into low‐ and high‐risk groups, and the Kaplan‐Meier plots with the Log‐rank test were used to compare the survival rate between the two groups. ROC analysis was used to evaluate the utility of the risk scores in predicting the OS of patients by identifying the area under the ROC curve (AUC) along with 95% CI. The AUC values ranged from 0.5 to 1.0, with a high AUC value indicating high sensitivity and specificity.

Additionally, the potential relationships between DNA methylation and the expression level of the corresponding genes were assessed by using the Spearman correlation analysis. Enrichment analysis of Gene Ontology (GO) and Kyoto Encyclopedia of Genes and Genomes (KEGG) pathway were carried out via the clusterProfiler package in R for those genes that significantly related to the two genes. Gene set enrichment analysis (GSEA) was performed to identify associated biological processes and signalling pathway.^25^

### 2.3 Immunohistochemistry assay

Lower grade glioma tissues and adjacent normal tissues were fixed by using 10% formalin. Then, those tissues were dehydrated, and embedded in paraffin. Those tissue sections were incubated with GALNT9 rabbit polyclonal antibody (1:100, Sanying) overnight. After washing with PBS thrice, those sections were incubated with secondary antibodies conjugated to horseradish peroxidase labelled polymers. Finally, those sections were counterstained with haematoxylin.

## 3 RESULTS

In this study, we tried to explore novel OS‐related DNA methylation in LGG patients to help distinguish potential malignancy, to guide clinical use of more reasonable and effective treatments. The entire workflow of this study is illustrated in Figure S1.

### 3.1 Derivation of prognostic DNA methylation biomarker

By performing univariate Cox proportional hazards regression analysis for DNA methylation level data derived from the training cohort (Table 1), a total of 26,183 DNA methylation sites that related to OS (*p* < 0.001, bootstrap > 90) were screened. Then these candidate methylation markers were used to carry out multivariate Cox regression analyses. At last, a combination model of two prognostic survival‐related DNA methylation sites was selected as the survival predictor. The combinatorial prognostic risk score was calculated for each patient based on their estimated regression coefficients. The risk score = (−6.733 × *β* value of cg00390143) + (−7.902 × *β* value of cg19598875). The genes corresponding with these two sites were *GALNT9* (polypeptide N‐acetylgalactosaminyltransferase 9) and *TMTC4* (transmembrane O‐mannosyltransferase targeting cadherins 4), respectively. The related information of these two DNA methylation sites are shown in Table S1.

**FIGURE 1:**
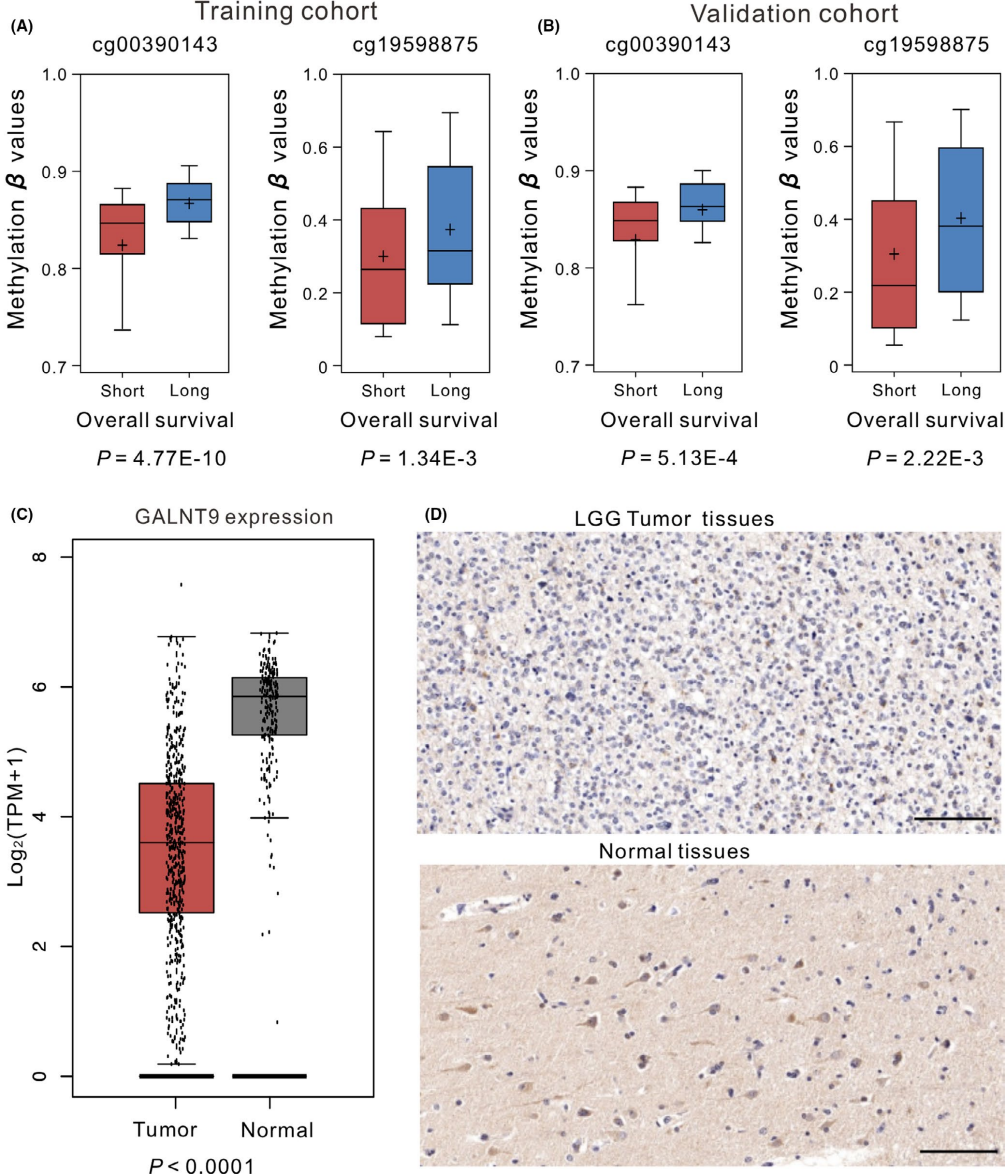
OS and methylation levels of patients with short survival (OS <3 years) and long survival (OS >3 years) in the training (A) and validation (B) cohorts. (C) The expression of genes in LGG. (D) Representative images of *GALNT9* expression in LGG tissues and corresponded normal tissues examined by immunohistochemistry. Scale bar = 100 μm. OS, overall survival

Meanwhile, the Mann‐Whitney *U* test was adopted to compare the differences of DNA methylation level in short (<3 years) and long (>3 years) survival groups. The results showed that long‐term survival patients exhibited significantly higher DNA methylation level (*p* < 0.001, Figure 1A,B), which was in accordance with the previous results of multivariate Cox regression analysis. Further analysis found that GALNT9 was significantly lower expression in LGG tissues (*p* < 0.001, Figure 1C), and immunohistochemistry analysis revealed that *GALNT9* gene was lowly expressed in LGG tissues (Figure 1D), which was consistent with the result in GEPIA database.

### 3.2 The association between the two‐CpG site DNA methylation signature and OS

**FIGURE 2:**
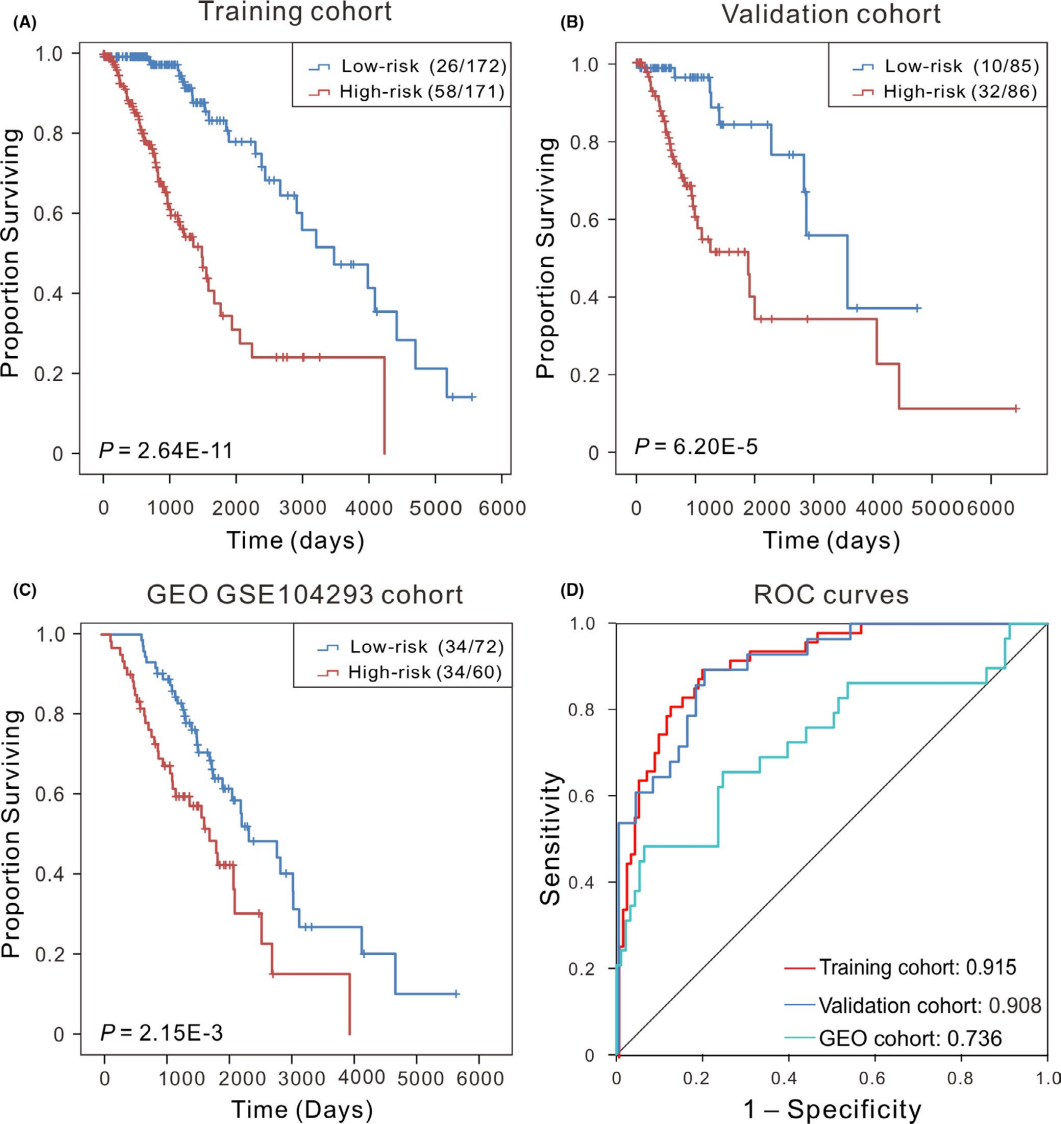
Kaplan‐Meier curves along with the Log‐rank test were used to visualize and compare the OS of the low‐risk vs. high‐risk groups in the training cohort (*N* = 353) (A) and the validation cohort (*N* = 171) (B). (C) Kaplan‐Meier survival curves showed the correlation between the signature and PFS in an independent cohort (GSE51547). Here, ‘low‐risk (26/172)’ refers to a total of 172 patients in the low‐risk group, among whom 26 were ‘death’, and ‘high‐risk (58/171)’ refers to a total of 171 patients in the high‐risk group, 58 of whom were ‘dead’ at the last status. (D) ROC curves showed the sensitivity and specificity of the signature in predicting the survival of patients in training cohort, validation cohort and GEO cohort, respectively. OS, overall survival

Cox regression analysis with the risk scores as a continuous variable showed that the two‐CpG site DNA methylation signature was significantly related to the OS of patients in both the training cohort (*p* = 2.92E‐18, HR: 2.72, 95% CI of HR: 2.17–3.40) and validation cohort (*p* = 2.29E‐9, HR: 2.189, 95% CI of HR: 1.69–2.83). With the signature and using the median risk score (−6.95) as the truncation value, patients were separated into high‐ and low‐risk groups. The Kaplan‐Meier curves along with the Log‐rank test were carried out to evaluate and compare the OS of patients in different groups. The results showed that this signature was able to significantly stratify patients into high‐ and low‐risk groups, and patients with higher risk score exhibited shorter OS (Figure 2A). To test this prognostic indicator, we calculated the risk score of patients from validation cohort, and observed similar results that high‐risk patients had worse OS (Figure 2B).

In order to analysis the specificity of the signature in predicting prognosis, time‐dependent ROC analysis was performed to calculate the AUC values by using a categorical variable for OS <3 years compared with the methylation signature. The AUC values were exceeding 0.90 (Figure 2D) in both training (0.915, 95% CI: 0.87–0.96) and validation cohorts (0.908, 95% CI: 0.84–0.97), revealing that this signature has good discriminatory ability in predicting prognosis. These results showed that the two‐CpG site DNA methylation signature has great potential in clinical applications, and can be served as a reliable prognostic biomarker.

### 3.3 Validation of the two‐CpG site DNA methylation signature in an independent cohort

To further assess the prognostic potential of our signature, an additional data set was downloaded (GSE104293, *N* = 132).^26^ The Kaplan‐Meier analysis demonstrated that patients whose risk score larger than the median risk score in the training cohort had a significantly shorter progression‐free survival (PFS) than those with low‐risk score (median PFS 1298 vs. 1841 days, *p* < 0.01, Figure 2C). The AUC estimate was 0.736 (*p* = 1.07E‐4, 95% CI: 0.62–0.85) (Figure 2D), suggesting that our signature is also an effective predictor of prognosis for LGG patients in other independent cohorts.

### 3.4 The independence of our signature in the OS prediction from clinical and pathological factors

**FIGURE 3:**
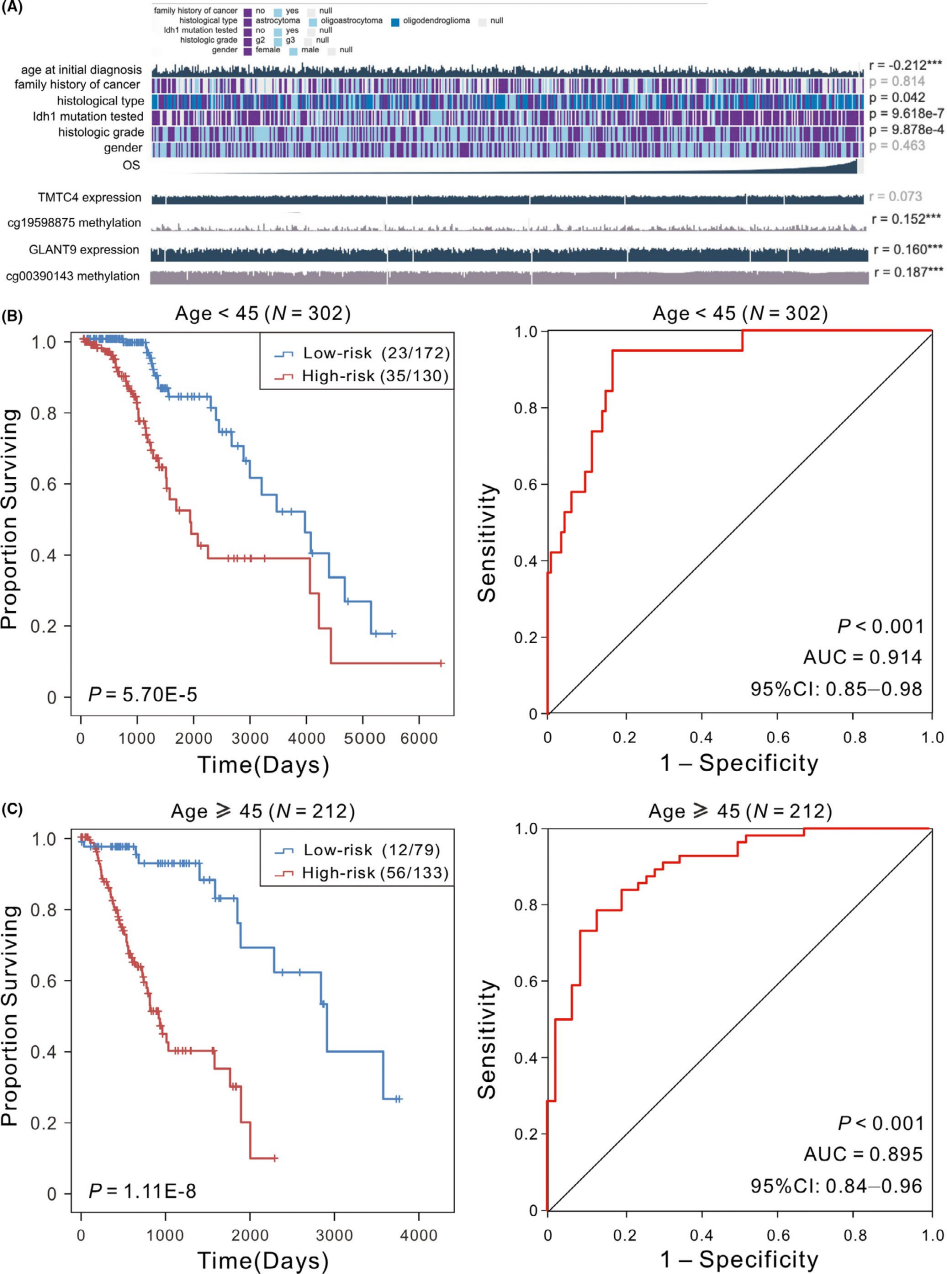
(A) The association between the clinicopathological factors and OS, * means *p* < 0.05, ** means *p* < 0.01, and *** means *p* < 0.001. (B) Kaplan‐Meier analysis with Log‐rank was carried out to assess the differences in OS of patients between the low‐risk and high‐risk in different age cohorts. (C) ROC curve analysis of the two‐CpG site DNA methylation signature was performed to demonstrate the sensitivity and specificity in patients’ OS prediction. OS, overall survival

Clinical factors such as gender, age, tumour grade, family history of cancer or IDH mutation status can affect patients’ survival.^27^ To analyse the independence and applicability of our signature, we regrouped the patients based on different clinicopathological features. Firstly, age is one of the greatest single predictor of cancer risk, and studies have shown that epigenetic age accelerates in precancerous tissue.^28^ Here, we found a significant negative correlation between the age of initial diagnosis and OS (*p* < 0.001, Figure 3A), and we divided the patients into two subgroups (younger than 45 years vs. 45 years or older). The Kaplan‐Meier analysis demonstrated that the OS of patients in the high‐risk group was much worse compared with patients in low‐risk group (*p* < 0.001), and ROC analyses confirmed that our signature had high predictive performance (AUC = 0.914 and 0.895, Figure 3B,C). Secondly, previous studies have shown that there are gender differences in both incidence and outcome of glioma patients, and the morbidity and mortality are higher for males than for females.^27^ Then regrouping was performed based on patients’ gender in our experiment. The results showed that patients with low‐risk scores had significantly (*p*  <  0.001) longer OS with an AUC value >0.90 (Figure S2), suggesting that our signature is independent of patients’ gender. As we all know that the malignant degree of glioma varies with different WHO grades. Although there were distinct differences in outcome in terms of disease progression, it has significant differences (*p*  <  0.001) in OS of patients between high‐ and low‐risk groups, and the AUC values for G2 and G3 cohorts were 0.878 and 0.912, respectively (Figure S3). Meanwhile, LGG are composed of astrocytoma, oligodendroglioma and mixed oligoastrocytoma, patients with astrocytomas generally have worse outcomes than patients with oligodendrogliomas.^29^ Here, irrespective of histologic subtype, our signature does a good job of distinguishing between low‐ and high‐risk groups, and patients in high‐risk group tend to have worse OS (*p*  <  0.001, Figure S4).

Isocitrate dehydrogenase mutation status is a known prognostic indicator of LGG, and compared with IDH1 wild‐type, patients with IDH mutation show a favourable survival outcome.^6^ Here, we found that AUC values of IDH status were 0.750 and 0.668 in TCGA cohort and another GEO data set (GSE10850, *N* = 185), respectively, indicating that the two‐CpG marker may have higher performance than IDH status in predicting the OS. Then we investigated whether our signature be used to distinguish survival risk of patients with different IDH mutational status. We found that our signature could distinguish the patients with low or high risks whether they had IDH mutations or not (Figure S5). Moreover, patients were divided into subgroups according to radiation therapy given or not, and the results found there is no association between the predictive performance of our signature and whether a patient received adjuvant radiation therapy (AUC > 0.9, Figure S6). In addition, family history is also an important risk factor for glioma, there is a significant increase in the risk of glioma in the first degree relatives of glioma patients.^30^ Here, 132 patients had family history of cancer. Regardless of family history, our signature is useful in distinguishing patients into subgroups with distinct outcomes (Figure S7).

In addition, stratified analyses were carried out, using two‐CpG site signature risk score and age or gender or WHO grade or histology subtype or IDH mutation status as covariates, respectively. The results showed that the two‐CpG site signature, the two‐CpG site signature together with clinical factors as covariates in the regression model had no significant differences (Table S2), suggesting that the two‐CpG site signature may be used as an independent predictor.

All these results indicated that our two‐CpG site DNA methylation signature can provide a better reference for risk stratification of patients, indicating that our signature was an independently applicable prognostic predictor of survival in patients with LGG. The results of Kaplan‐Meier and ROC analyses were shown in Table 2.

**TABLE 2:** Results of Kaplan‐Meier and ROC analyses based on different regrouping methods

Regrouping factors	Group	Sample size	HR (95% CI)	Kaplan‐Meier *p*‐value	AUC	95% CI
Gender	Male	285	2.832 (2.22–3.60)	2.85E‐10	0.925	0.88–0.87
	Female	229	2.238 (1.75–2.86)	2.00E‐05	0.902	0.74–0.97
Age at diagnosis	<45	302	2.145 (1.66–2.78)	5.70E‐05	0.914	0.85–0.96
	≥45	212	2.538 (1.99–3.24)	1.11E‐08	0.895	0.83–0.96
WHO grade	G2	248	2.263 (1.52–3.37)	4.30E‐05	0.878	0.79–0.97
	G3	265	2.122 (1.76–2.56)	2.28E‐07	0.912	0.86–0.96
Histologic subtype	Astrocytoma	194	2.587 (1.97–3.40)	6.08E‐07	0.954	0.91–0.99
	Oligodendroglioma	189	2.169 (1.54–3.05)	4.33E‐04	0.841	0.75–0.93
	Mixed glioma	131	2.366 (1.77–3.16)	2.84E‐04	0.936	0.86–1.00
IDH1 mutation	Yes	91	1.919 (1.13–3.25)	1.03E‐02	0.933	0.81–1.00
	No	34	3.258 (1.48–7.15)	8.46E‐03	1.00	1.00–1.00
Radiation therapy	Yes	277	2.081 (1.71–2.54)	7.31E‐09	0.915	0.87–0.96
	No	169	3.414 (2.72–5.36)	8.00E‐03	0.879	0.77–0.97
Family history of cancer	Yes	132	2.909 (2.01–4.21)	7.50E‐05	0.976	0.94–1.00
	No	210	2.379 (1.81–3.12)	1.50E‐05	0.903	0.84–0.97

### 3.5 Comparison of our signature with other known prognostic markers

**FIGURE 4:**
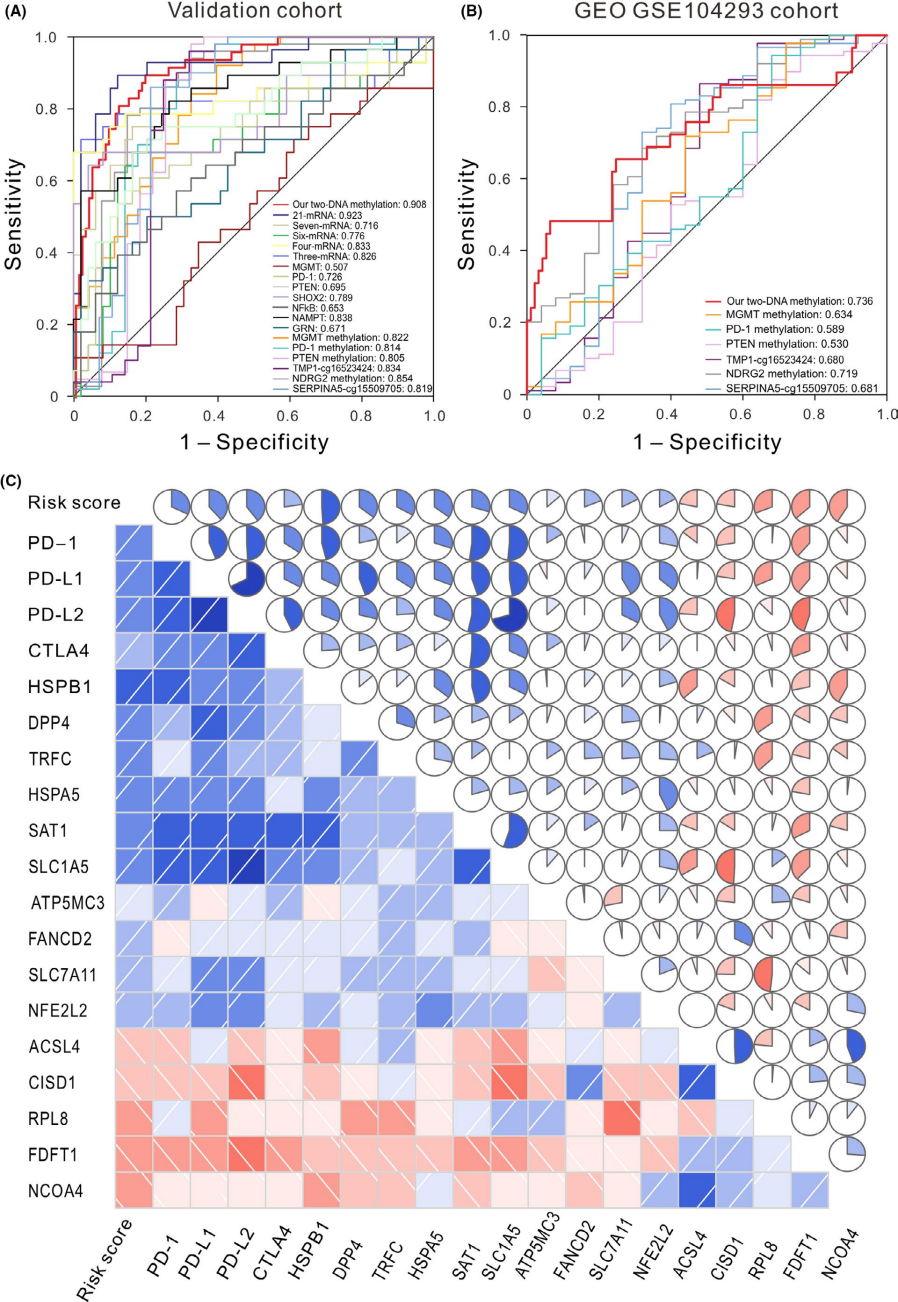
ROC curves delineate the sensitivity and specificity of our signature and other known markers in predicting the OS of patients from TCGA validation data set (A) and another independent cohort (B). (C) Correlation analyses between our two‐CpG site DNA methylation signature, known immune‐checkpoint genes, and ferroptosis regulator genes. Upper triangle: circle symbols represent the one‐to‐one correlation coefficient; each correlation coefficient is shown by fill area and intensity of shading, which increases uniformly as the correlation value moves away from 0; blue for positive correlation, red for negative correlation. Lower triangle: blue and slashes from bottom left to top right indicate a positive correlation between two variables. Conversely, red and slashes from the top left to the bottom right indicate a negative correlation between variables. The darker the colour, the higher the saturation, indicating the greater the correlation of two variables. OS, overall survival

Recently, several prognostic markers for LGG have been identified. For instance, NF‐κB has been identified as an independent predictor of both OS and malignant PFS in LGGs.^31^ PD‐1 promoter methylation has been showed to be a prognostic factor in diffuse LGG IDH mutations patients.^32^ Song et al. found that the expression levels of 21 mRNAs can be combined as a prognostic biomarker for LGG patients.^5^ To compare our prognostic signature with known models, we carried out time‐dependent ROC curve analysis in our signature and other models in both TCGA validation and GEO data set. The results showed that our signature has a higher AUC value than almost all the other known models including all clinical factors, mRNA markers, DNA methylation markers in both TCGA validation cohort (Figure 4A and Table S3) and GEO cohort (Figure 4B and Table S4), indicating that our signature had a satisfactory efficiency in predicting the OS of LGG patients.

### 3.6 Association of the signature with ICB immunotherapy‐related signature and ferroptosis regulator genes

Recently, cancer immunotherapy using immune‐checkpoint blockade (ICB) that targets programmed cell death 1 (PD‐1), programmed cell death‐ligand 1 (PD‐L1), programmed cell death‐ligand 2 (PD‐L2) and cytotoxic T‐lymphocyte‐associated protein 4 (CTLA‐4) provides significant clinical benefits in cancer.^33^ Ferroptosis is a newly discovered type of cell death related to cancer, and play important roles during cancer progression and treatment.^34^ Here, to further investigate the possible role of our methylation signature in ICB treatment and cancer progression, we conducted a one‐to‐one correlation analysis between these known immunotherapy‐related genes, ferroptosis regulator genes and our two‐CpG site DNA methylation signature. Results showed that immune‐checkpoint molecules *PD*‐*1*, *PD*‐*L1*, *PD*‐*L2* and *CTLA*‐*4* were coexpressed (*p* < 0.001), our signature was significantly positively related to *PD*‐*1*, *PD*‐*L1*, *PD*‐*L2* and *CTLA*‐*4* (*p* < 0.001 and *r* = 0.320, 0.383, 0.395 and 0.225, respectively) (Figure 4C and Figure S8). For ferroptosis regulator genes, the DNA methylation signature was significantly positively correlated with HSPB1, HSPA5, DPP4, TFRC, SAT1, SLC1A5, FANCD2, ATP5MC3, SLC7A11, NFE2L2 (*p* < 0.001), while significantly negatively correlated with ACSL4, CISD1, RPL8, FDFT1, NCOA4 (*p* < 0.001) (Figure 4C). These results imply that the two‐CpG site DNA methylation signature may play a role in measures of responsiveness to ICB immunotherapy and oncogenesis and development.

### 3.7 GO and KEGG analysis of the genes associated with two genes in our signature

**FIGURE 5:**
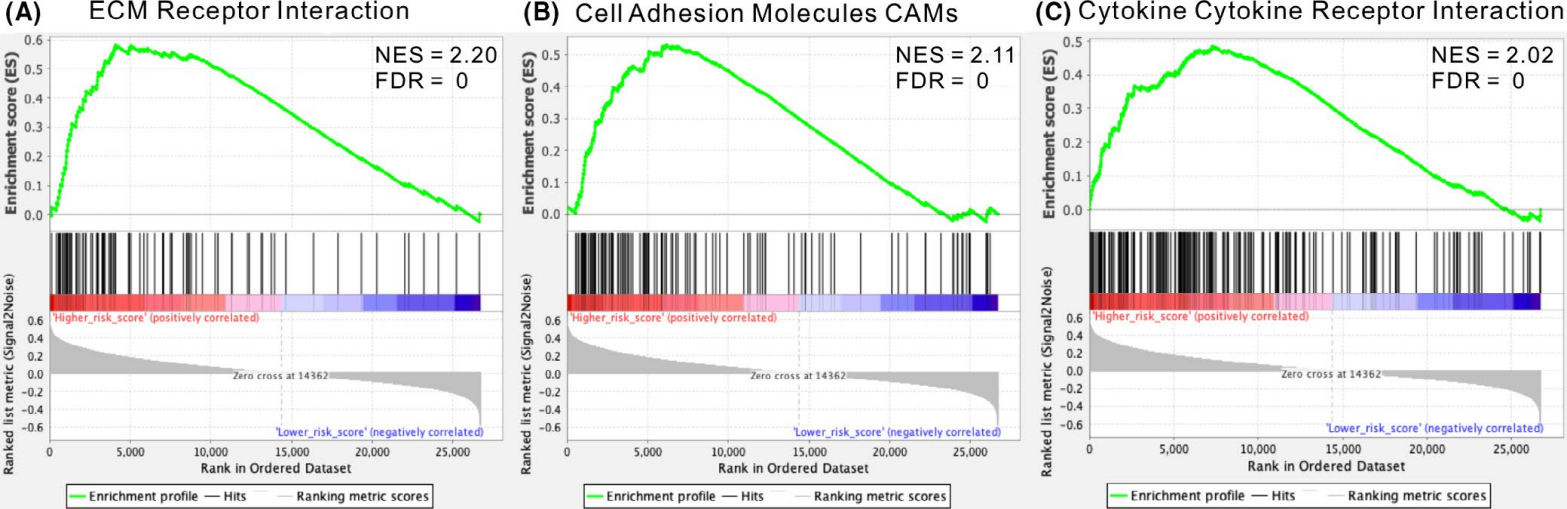
Gene set enrichment analysis show biological pathways and processes associated with risk score in the TCGA cohort. (A) ECM receptor interactions pathway, (B) cell adhesion molecules CAMs pathway, and (C) cytokine cell receptor interactions pathway

Correlation analysis showed that 2736 genes were significantly related to *GALNT9* and *TMTC4*, among which 688 genes were significantly positively correlated with these two genes (*p* < 0.001). GO enrichment analysis displayed that these screened genes mainly enriched in intracellular signal transduction, regulation of biological process and response to stimulus. The enrichment analysis of KEGG pathway indicated that these selected genes were mainly concentrated in metabolic pathway, phagosome, MAPK signalling pathway, B‐cell receptor signalling pathway, Rap1 signalling pathway (Figure S9A). Reactome pathway analysis showed a predominance of genes involved in immune system, neutrophil degranulation, signalling by Rho GTPases or interleukins, and cell cycle (Figure S9B). Gene set enrichment analysis of genes significantly differentially expressed in the high‐risk and low‐risk groups showed that several cancer‐related pathways, including ECM receptor interactions, cell adhesion molecules CAMs and cytokine cell receptor interactions, were enriched in the high‐risk group, implying that the signature may be involved in metastasis‐related pathways (Figure 5).

## 4 DISCUSSION

DNA methylation is one of the most common epigenetic events in disease.^35^ In recent years, abnormal DNA methylation has been shown to play an important role in a number of diseases,^36^ particularly cancers.^37^ Aberrant methylation can be used as biomarker for clinical diagnosis, prognosis and decisions of different cancers.^19^, ^38^ LGGs are heterogeneous and invasive in nature, and the strategies used for clinical management are highly variable. Effective prognostic biomarkers can stratify LGG patients according to risk at initial diagnosis and contribute to clinical treatment options. Here, we have developed a two‐CpG site DNA methylation prognostic signature that can be used to classify LGG patients into high‐ and low‐risk according to the survival. We tested the signature in validation and a GEO cohorts.

An ideal prognostic biomarker is that it should be independent of clinicopathological prognostic factors currently in use and can also efficiently differentiate risk stratification in other independent cohorts. For instance, although amplification or mutation of EGFR is widely believed to be an indicator of poor survival, some studies have failed to confirm this conclusion.^39^, ^40^ Here, according to different clinicopathological characteristics, patients were regrouped, and the results revealed that our two‐CpG site DNA methylation signature was independent of other clinical factors like age, WHO grade, family history of cancer and IDH mutation status. Meanwhile, in another independent cohort, our signature was superior to other known prognostic markers, including mRNA, and DNA methylation, and statistical comparison was carried out using Z‐test. Therefore, our signature was an independent applicable prognostic biomarker with high predictive performance, which may be priority of clinical perspective.

Furthermore, studies have shown that epigenetic changes can alter gene expression, and epigenetic inactivation of tumour suppressor genes is associated with tumorigenesis of various cancers.^41^ Here, we found that the expression of *GALNT9* was significantly (*p* < 0.001) positive related to its methylation level, and the other gene show negative significant positive correlation (*p* = 0.0017) between the expression and its methylation level (Figure S10A,B). Immunohistochemical analysis showed that GALNT9 gene was lower expressed in LGG tissues than adjacent normal tissues. Although we did not give accurate statistical results due to the limitation of samples, we found that GALNT9 was significantly lower expression in LGG, glioblastoma (GBM), cervical Cancer (CESC), colon Cancer (COAD), kidney chromophotoma (KICH), kidney papillary cell carcinoma (KIRP), thyroid cancer (THCA), and higher expression in thymoma (THYM) than in normal tissue in the public database GEPIA (Figure S10C). TMTC4 was higher expression in LGG, glioblastoma (GBM), bile duct cancer (CHOL), COAD, large B‐cell lymphoma (DLBC), prostate cancer (PRAD), rectal cancer (READ), and THYM than in normal tissue (Figure S10D). For our two DNA methylation sites, studies have showed that their corresponding genes may play a key role in cancer development.^42^ *GALNT9* is expressed specifically in the brain, with highest expression in the cerebellum.^43^ Pangeni *et al* ^42^ have found GALNT9 is often epigenetically dysregulated in breast tumours that metastasis to the brain, and may be involved in the progression of primary breast tumours to brain metastases. GALNT9 was significantly higher expression in high‐grade serous ovarian cancer,^44^ and expression of GALNT9 was associated with OS, can be serve as a prognostic marker for personalized therapy of neuroblastoma.^45^ TMTC4 is expressed in the cytoplasm of most tissues, and is ubiquitously expressed in the brain. TMTC4 is mainly enriched in the endoplasmic reticulum, and plays a role by regulating Ca^2+^ dynamics and protein folding reactions.^46^ Although the functional mechanism of these two genes in LGG remains to be further explored, and supplemental basic experiments are still warranted, the DNA methylation of these two sites might also be suitable as biomarkers for prognostic prediction in LGG.

Complex tumour microenvironment is known to regulate tumour development and growth in gliomas, and tumour‐infiltrating immune cells are an important part of this microenvironment.^47^ Studies have shown that a high numbers of intratumoral effector T cells is significantly associated with a better survival in glioma.^48^ Here, we find that the expression level of *GALNT9* and *TMTC4* was significantly positively related to the infiltration level of B cells, CD4+ and CD8+ T cells lymphocytic infiltration (*p* < 0.001), and negatively correlated with macrophages (*p* < 0.001) (Figure S11). Meanwhile, the two‐CpG site DNA methylation signature was significantly related to ICB immunotherapy‐related signature and ferroptosis regulator genes. Moreover, glioma CpG island methylator phenotype (G‐CIMP) is known to be useful for predicting patient outcome, and G‐CIMP + tumours were closely related to IDH mutation and had a favourable prognosis.^49^ Here, we tested the prognostic interrelationship of the two‐CpG site signature with known G‐CIMP. The results found that patients with G‐CIMP+had a favourable prognosis, and patients with G‐CIMP‐ have significantly higher two‐CpG site signature risk scores (Figure S12). These results suggest that these two genes may be linked to cancer progression, tumour immunity as well as G‐CIMP, and have the potential application in tumour immunotherapy in the future.

Additionally, the univariate Cox regression, the Kaplan‐Meier and ROC analyses for the two individual methylation sites were carried out, and the results showed that each DNA methylation sites could also distinguish patients with different risk, but the predictive performances were not as high as their combination in both the training and validation cohorts (Figures S13 and S14), suggesting that single DNA methylation site may play a role in the prognostic prediction, while the combined methylation signature might provide better potential for achieving more sensitive and specific prognostic value in LGG patients. To our knowledge, the prognostic value of this two DNA methylation signature in LGG has not been previously reported yet. Therefore, the present study provides a new insight that a combination of epigenetic biomarkers may improve risk stratification and survival prediction in LGG patients.

In conclusion, we identified and validated a two‐CpG site DNA methylation signature by analysing TCGA and GEO DNA methylation data. The signature was significantly related to the OS of patients with LGG, and could distinguish the prognosis of patients based on survival risk. The Kaplan‐Meier and ROC analyses indicated that the signature had independent prognostic features and high prognostic accuracy. Further analysis indicated that this signature was significantly correlated with ICB immunotherapy‐related signatures and ferroptosis regulator genes. And the expression pattern and functional analysis showed that the genes corresponding to the two sites included in the model were associated with the level of immune infiltration, and involved in MAPK and Rap1 signalling pathway, suggesting that these two genes may have the potential application in tumour immunotherapy. The signature would be helpful to improve risk stratification and provide a more accurate assessment of clinical individualization. In addition to their use as a biomarker, these DNA methylation sites require further detailed study to determine the mechanism and elucidate how they affect patients’ outcomes.

## CONFLICT OF INTEREST

All authors declare that they have no conflicts of interests.

## AUTHOR CONTRIBUTIONS

**Wenna Guo:** Conceptualization (lead); formal analysis (lead); investigation (lead); resources (lead); writing – original draft (lead). **Shanshan Ma:** Data curation (lead); formal analysis (supporting); investigation (supporting); resources (supporting); writing – review and editing (supporting). **Yanting Zhang:** Data curation (supporting); investigation (supporting); visualization (supporting); writing – review and editing (supporting). **Hongtao Liu:** data curation (supporting); supervision (supporting); visualization (supporting). **Ya Li:** Formal analysis (supporting); investigation (supporting). **Ji‐Tian Xu:** Validation (supporting); writing – review and editing (supporting). **Bo Yang:** Project administration (supporting); Supervision (lead); validation (lead); writing – review and editing (supporting). **Fangxia Guan:** Funding acquisition (equal); project administration (lead); writing – review and editing (supporting).

## Supporting information

Supplementary Material

## Data Availability

The information of this study here is obtained from the TCGA (https://portal.gdc.cancer.gov/), the NCBI GEO (https://www.ncbi.nlm.nih.gov/geo/).
