# Supplementary material for: Genome‐wide methylomic analyses identify prognostic epigenetic signature in lower grade glioma
Source: J Cell Mol Med. 2021 Dec 11;26(2):449–61. doi: 10.1111/jcmm.17101 (PMC8743658; doi:10.1111/jcmm.17101)
Supplement: Supplementary file 1 — Supplementary Material [file JCMM-26-449-s001.pdf]

Table S1 Two significantly survival-related methylation sites in training dataset.

Probe ID	Chromosomal location	Gene symbol	CGI coordinate	Feature type	*P* value[a]	Coef.[b]	*P* value[b]
cg00390143	chr12:132265953–132265954	*GALNT9*	chr12:132265952–132266198	Island	5.69E-13	–6.733	2.16E-08
cg19598875	chr13:100673938–100673939	*TMTC4*	chr13:100674075–100675469	N_Shore	1.36E-09	–7.902	5.22E-08

[a]. in univariate Cox regression analysis;
[b]. in multivariate Cox regression analysis;

Table S2 Results for Cox regression models of the two-CpG site signature and clinical factors as covariates.

Variables	Univariate Cox model			Multivariate Cox model		
	HR	95% CI	*P*-value	HR	95% CI	*P*-value
Two-CpG signature	2.38	2.02-2.80	<0.0001	2.354	1.97-2.81	<0.0001
Age	1.059	1.04-1.07	<0.0001	1.053	1.04-1.07	<0.0001
Two-CpG signature	2.38	2.02-2.80	<0.0001	2.479	2.01-2.93	<0.0001
Gender	0.925	0.65-1.32	0.665	0.657	0.45-0.95	0.024
Two-CpG signature	2.38	2.02-2.80	<0.0001	2.153	1.82-2.55	<0.0001
WHO grade	3.277	2.22-4.83	<0.0001	2.209	1.47-3.31	<0.0001
Two-CpG signature	2.38	2.02-2.80	<0.0001	2.373	2.01-2.79	<0.0001
Histologic subtype	0.761	0.60-0.97	0.026	0.915	0.73-1.15	0.448
Two-CpG signature	2.38	2.02-2.80	<0.0001	2.372	1.59-3.53	<0.0001
IDH status	5.521	2.06-14.79	0.0006	4.108	1.49-11.33	0.006

Table S3 The ROC results of two-DNA methylation signature and other known biomarkers in the TCGA validation cohort.

Signature	AUC	95% CI of AUC	*P* value[a]	Type	*P* value[b]	Ref
Two-DNA methylation	0.908	0.84–0.97	2.72E-09	Methylation		This study
21-mRNA	0.923	0.86–0.99	7.63E-10	Protein coding	0.624	[1]
Seven-mRNA	0.716	0.59–0.85	1.66E-03	Protein coding	0.005	[2]

Six-mRNA	0.776	0.65–0.90	6.30E-05	Protein coding	0.037	[3]
Four-MRNA	0.833	0.71–0.95	1.00E-06	Protein coding	0.137	[4]
Three mRNA	0.826	0.70–0.94	2.00E-06	Protein coding	0.122	[5]
MGMT	0.507	0.37–0.64	0.924	Protein coding	< 0.001	[6]
PD-1	0.726	0.60–0.85	1.03E-03	Protein coding	0.005	[7]
PTEN	0.695	0.62–0.77	3.00E-05	Protein coding	< 0.001	[8]
NFkB	0.653	0.52–0.79	0.026	Protein coding	< 0.001	[9]
SHOX2	0.789	0.67–0.91	2.70E-05	Protein coding	0.043	[10]
SERPINA5	0.841	0.75–0.93	7.20E-07	Protein coding	0.125	[11]
TIMP1	0.835	0.72–0.95	1.05E-06	Protein coding	0.140	[11]
NAMPT	0.838	0.74–0.94	8.95E-07	Protein coding	0.121	[12]
GRN	0.671	0.54–0.80	0.013	Protein coding	0.001	[12]
SERPINE1	0.779	0.67–0.89	5.00E-05	Protein coding	0.025	[12]
six-CpG signature	0.947	0.88–1	7.01E-11	Methylation	0.802	[13]
MGMT	0.822	0.72–0.92	3.00E-06	Methylation	0.078	[6]
NDRG2	0.854	0.75–0.96	2.39E-07	Methylation	0.194	[14]
PTEN	0.805	0.67–0.93	9.00E-06	Methylation	0.079	[15]
PD-1	0.814	0.69–0.94	5.00E-05	Methylation	0.090	[7]
cg12434587	0.63	0.47–0.78	0.058	Methylation	0.001	[16]
cg12981137	0.695	0.56–0.83	0.004	Methylation	0.003	[16]
cg27151711	0.799	0.66–0.93	1.30E-05	Methylation	0.077	[11]
cg16523424	0.834	0.72–0.95	1.00E-06	Methylation	0.137	[11]
cg04791822	0.780	0.64–0.92	4.40E-05	Methylation	0.053	[11]
cg15509705	0.819	0.70–0.94	3.00E-06	Methylation	0.097	[11]
Gender	0.504	0.37–0.64	9.58E-01	Clinical factor	< 0.001	
Age	0.835	0.73–0.94	1.00E-06	Clinical factor	0.005	
Grade	0.607	0.48–0.74	0.118	Clinical factor	< 0.001	
IDH1	0.809	0.56–1.00	5.40E-02	Clinical factor	0.225	
Subtype	0.554	0.41–0.70	4.68E-01	Clinical factor	< 0.001	
Radiation therapy	0.581	0.44–0.72	2.76E-01	Clinical factor	< 0.001	
Family history of cancer	0.521	0.35–0.70	8.11E-01	Clinical factor	< 0.001	

[a]. in ROC analysis;

[b]. in the statistical comparison (Z-test) between AUC value of corresponding signature and the two-DNA methylation signature.

Table S4 The ROC results of two-DNA methylation signature and other known biomarkers in the GSE104293 validation cohort.

Signature	AUC	95% CI of AUC	*P* value[a]	Type	*P* value[b]	Ref
Two-DNA methylation	0.736	0.62–0.85	1.81E-04	Methylation		This study
six-CpG signature	0.686	0.56–0.81	0.064	Methylation	0.284	[13]
MGMT	0.634	0.50–0.76	0.042	Methylation	0.111	[6]
NDRG2	0.719	0.60–0.83	0.001	Methylation	0.145	[14]
PTEN	0.53	0.38–0.67	0.649	Methylation	0.137	[15]
PDCD1	0.589	0.45–0.72	0.176	Methylation	0.270	[7]
cg12434587	0.629	0.50–0.75	0.049	Methylation	0.031	[16]
cg12981137	0.641	0.51–0.77	0.031	Methylation	0.280	[16]
cg27151711	0.63	0.48–0.78	0.048	Methylation	0.130	[11]
cg16523424	0.68	0.54–0.81	0.006	Methylation	0.419	[11]
cg04791822	0.563	0.42–0.70	0.339	Methylation	0.015	[11]
cg15509705	0.681	0.54–0.82	0.006	Methylation	0.054	[11]
Gender	0.542	0.42–0.66	0.485	Clinical factor	0.011	
Age	0.529	0.40–0.65	0.629	Clinical factor	0.009	
Radiation therapy	0.558	0.44–0.68	0.34	Clinical factor	0.018	
MGMT status	0.551	0.43–0.67	0.401	Clinical factor	0.017	

[a]. in ROC analysis;

[b]. in the statistical comparison (Z-test) between AUC value of corresponding signature and the two-DNA methylation signature.

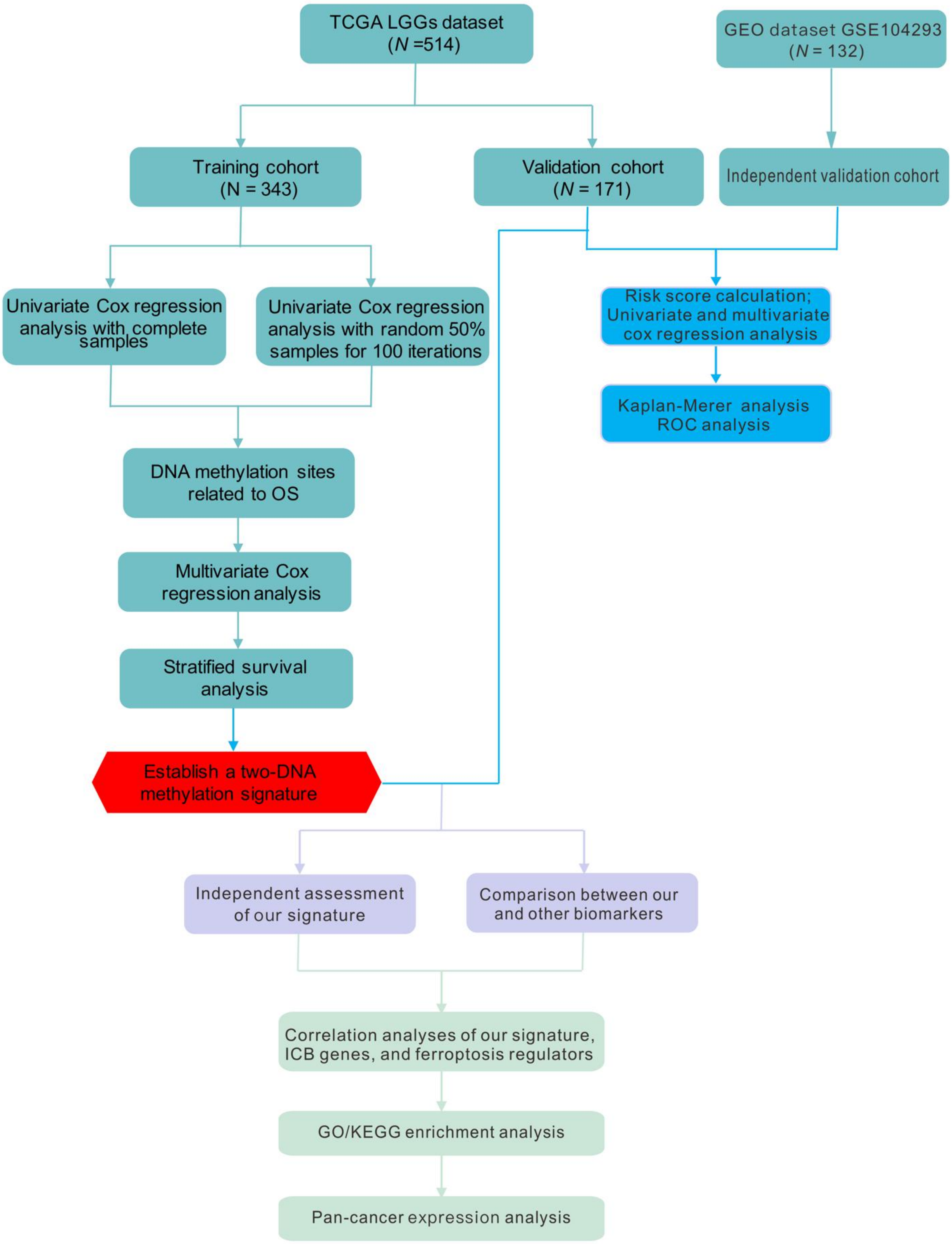

Figure S1. Workflow for the construction and validation of the DNA methylation prognostic signature.

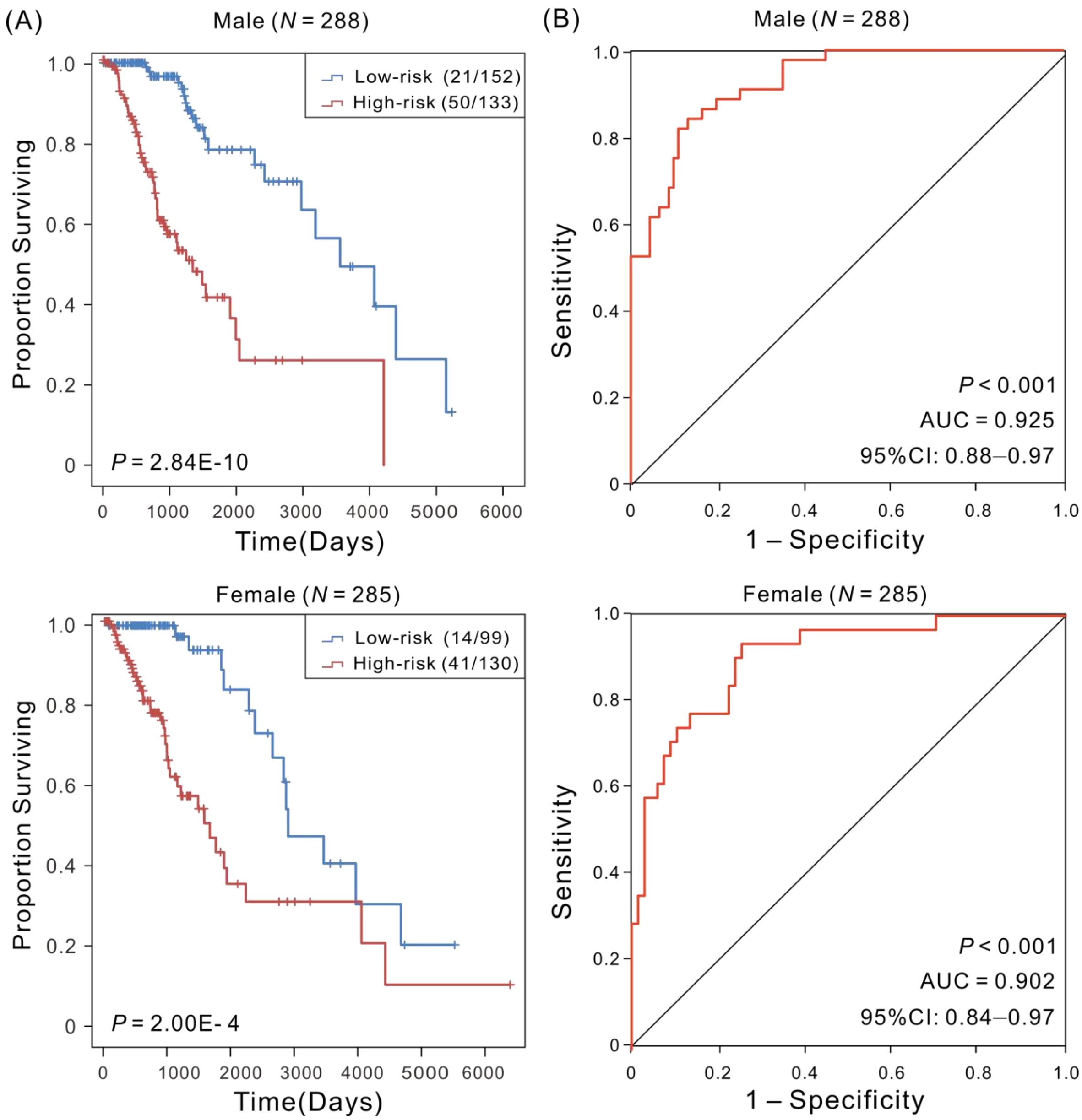

Figure S2. Kaplan–Meier and ROC analyses of LGG patients in different sex groups. **(A)** Kaplan–Meier estimates of the patients' OS for low- and high-risk patient, and the OS differences between two groups were determined by Log-rank test; **(B)** ROC curves show the sensitivity and specificity of the signature in predicting the OS of patients.

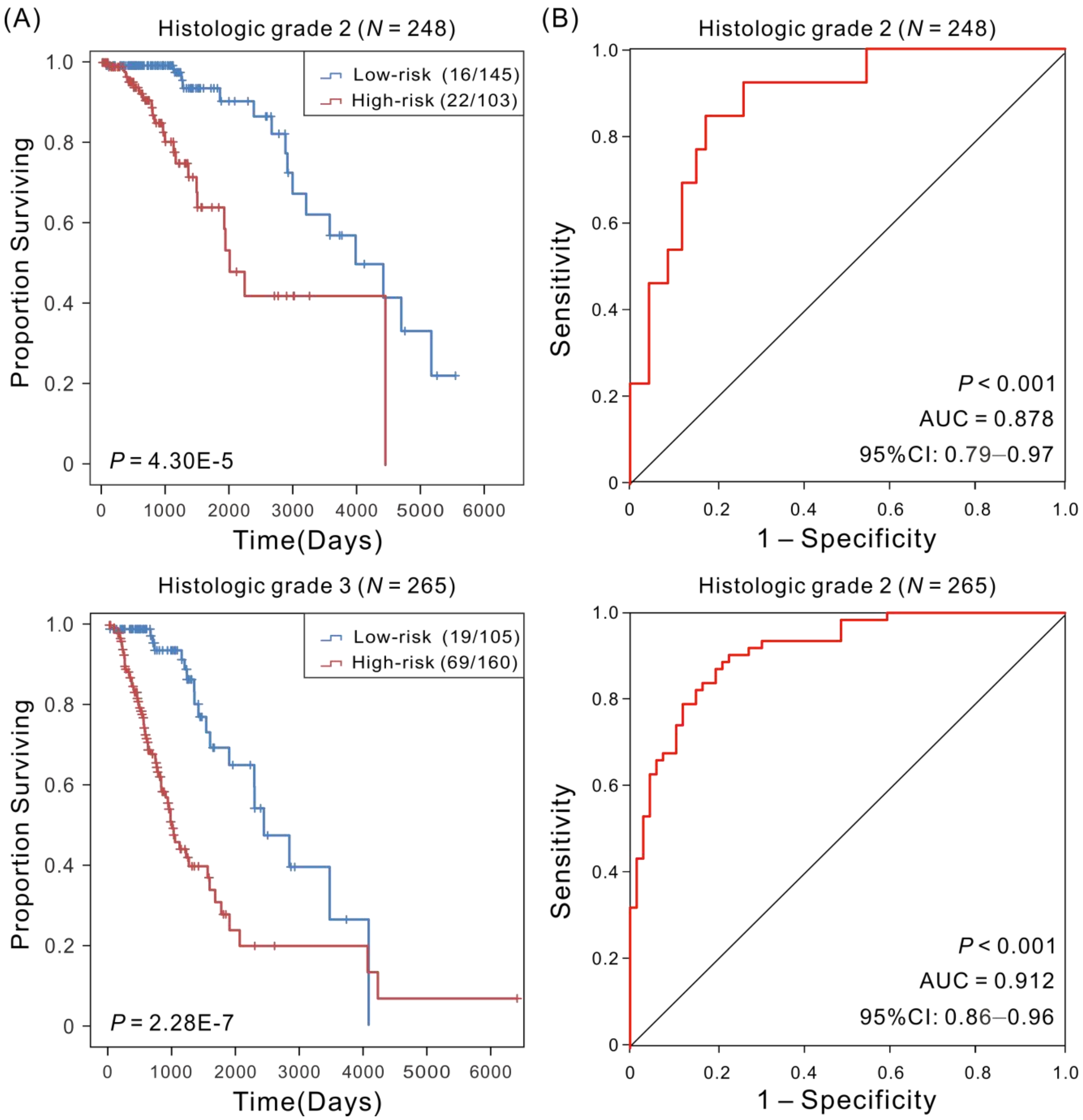

Figure S3. Kaplan–Meier and ROC analyses of LGG patients with different WHO grades. **(A)** Kaplan–Meier analysis with Log-rank test was performed to estimate the differences in OS between the low- and high-risk patients. **(B)** ROC curves of the signature were used to demonstrate the sensitivity and specificity in predicting the OS of patients.

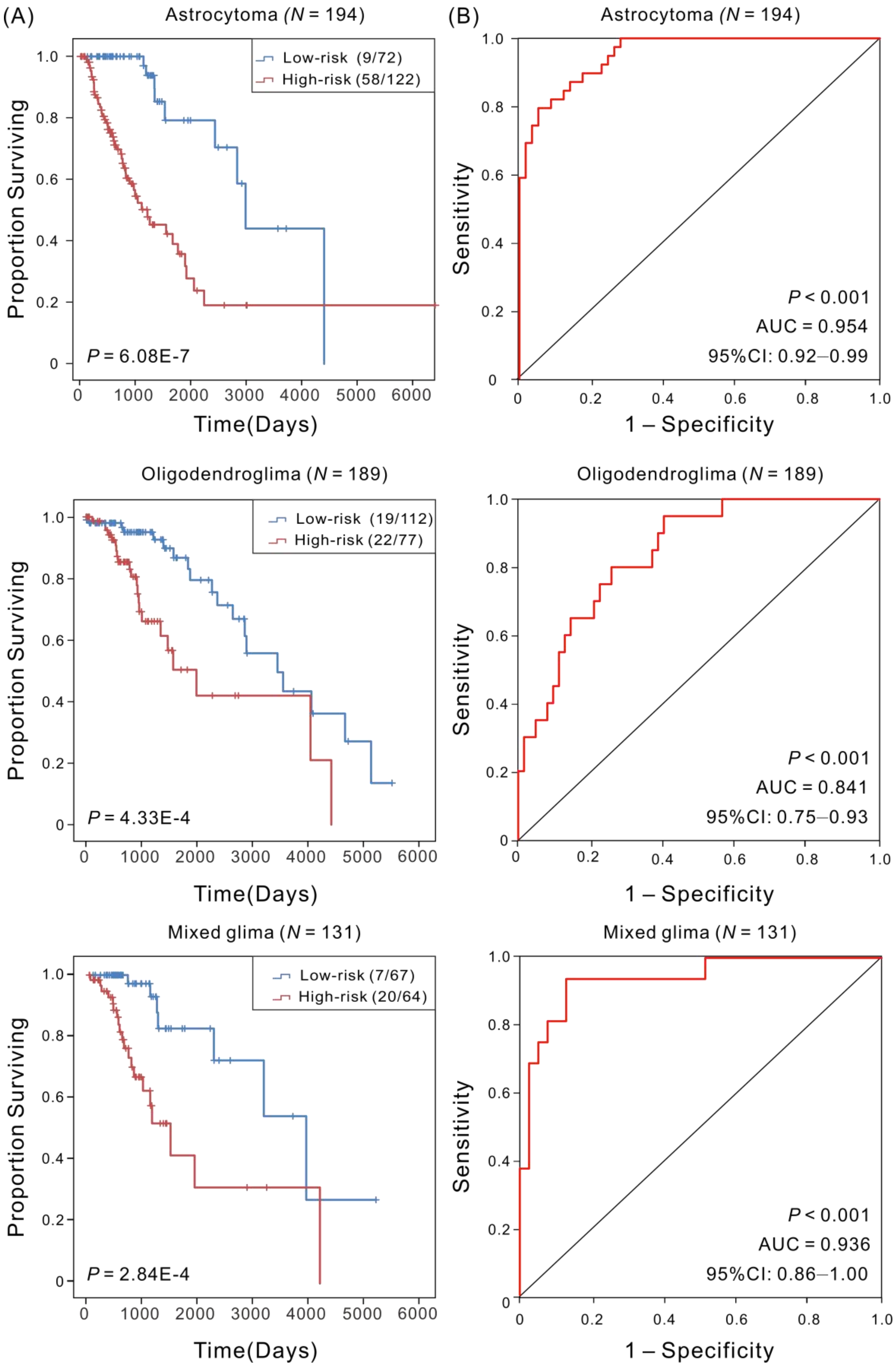

Figure S4. Kaplan–Meier and ROC analyses of LGG patients with different histologic. **(A)** Kaplan–Meier estimates of the patients' OS for low- and high-risk patient in different stage cohorts, and the OS differences between two groups were determined by Log-rank test; **(B)** ROC curves show the sensitivity and specificity of the signature in predicting the OS of patients.

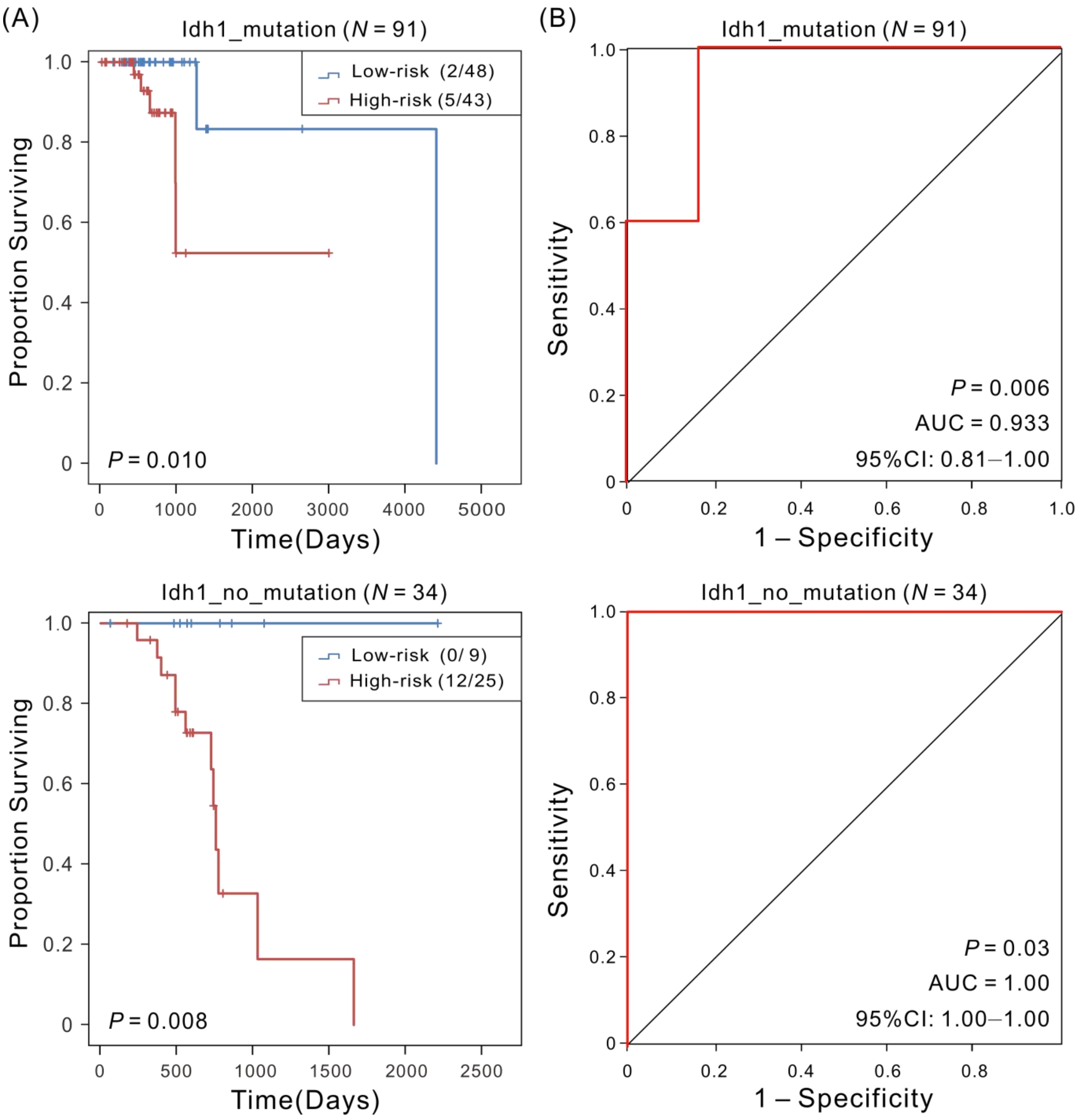

Figure S5. Kaplan–Meier and ROC analyses of LGG patients with IDH1muation and wild-type. (A) Kaplan–Meier analysis with Log-rank was performed to estimate the differences in OS between the low- and high-risk patients. **(B)** ROC curves of the signature were used to demonstrate the sensitivity and specificity in predicting the OS of patients.

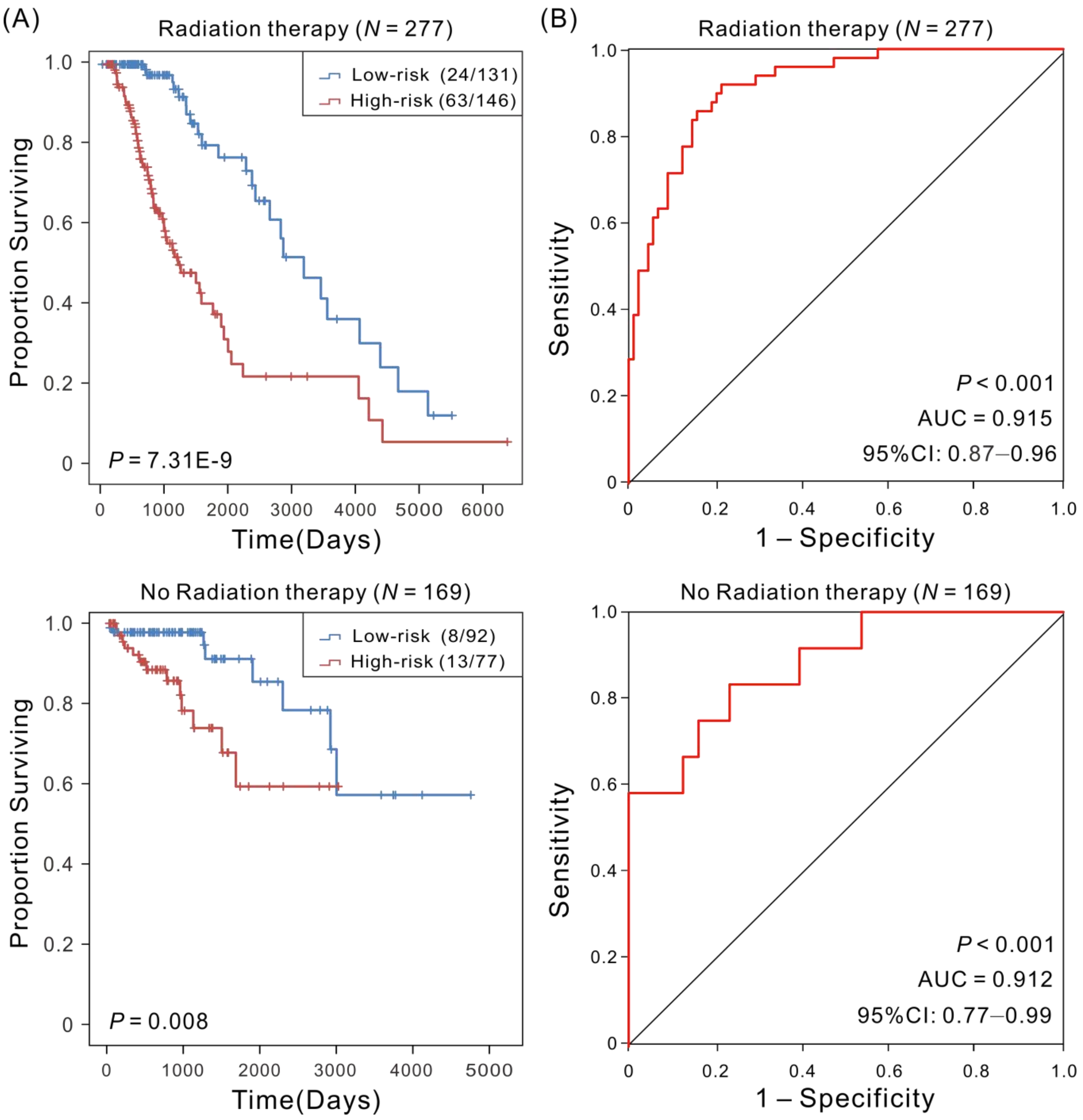

Figure S6. Kaplan–Meier and ROC analyses of LGG patients received adjuvant radiation therapy or not, respectively. **(A)** Kaplan–Meier analysis with Log-rank test was performed to estimate the differences in OS between the low- and high-risk patients. **(B)** ROC curves of the signature were used to demonstrate the sensitivity and specificity in predicting the OS of patients.

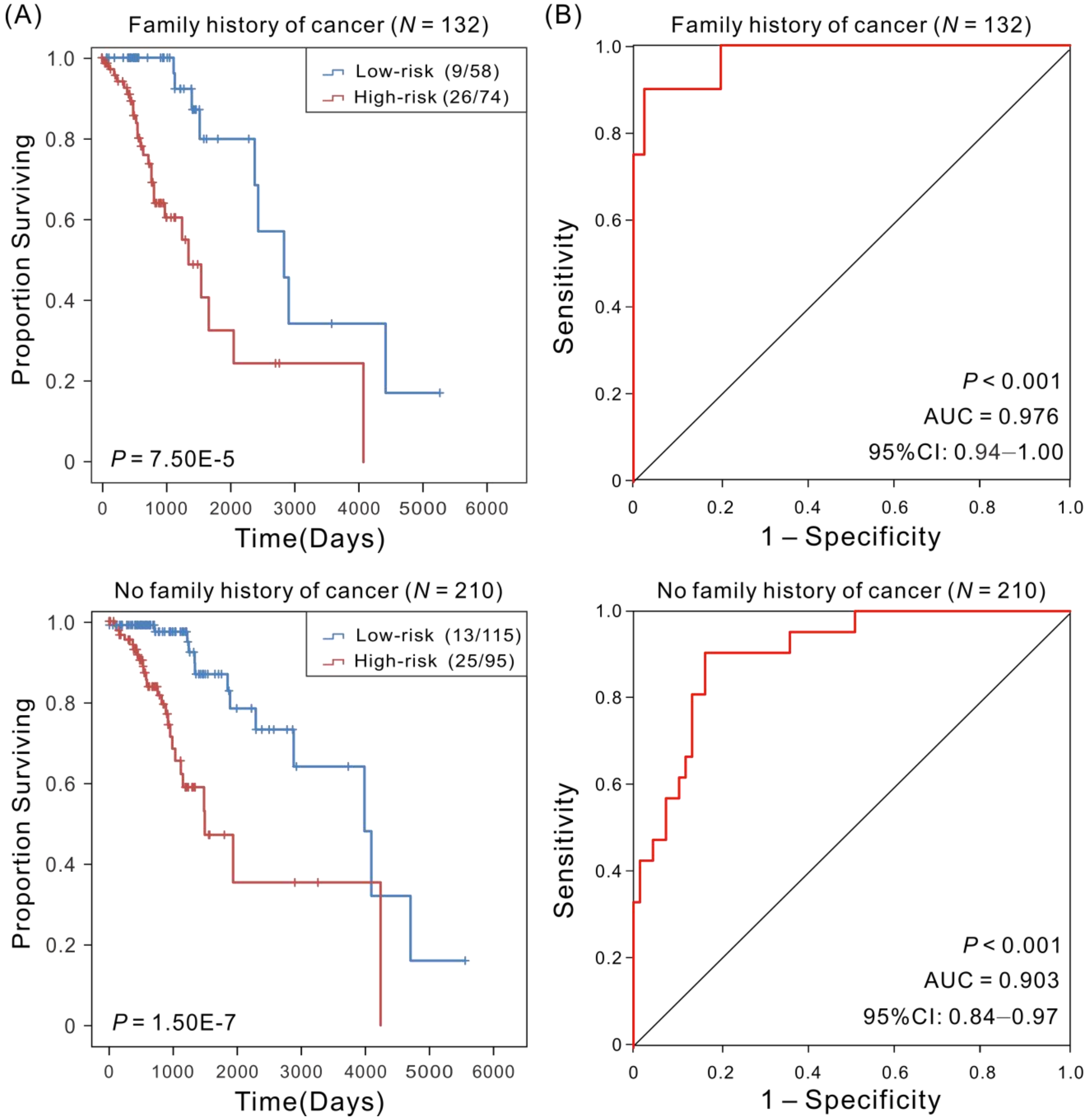

Figure S7. Kaplan–Meier and ROC analyses of LGG patients have family history of cancer. (A) Kaplan–Meier analysis with Log-rank was performed to estimate the differences in OS between the low- and high-risk patients. **(B)** ROC curves of the signature were used to demonstrate the sensitivity and specificity in predicting the OS of patients.

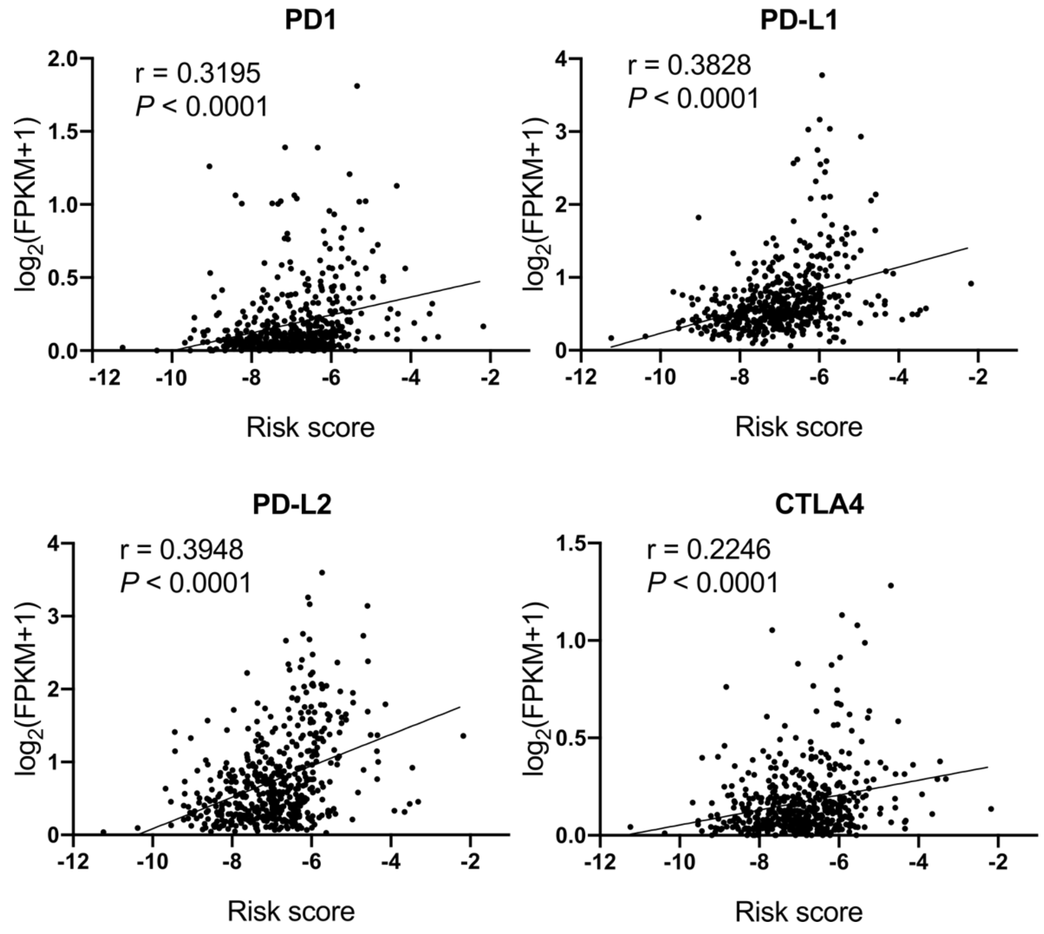

Figure S8. Correlation between two-DNA methylation risk score and ICB immunotherapy-related signature.

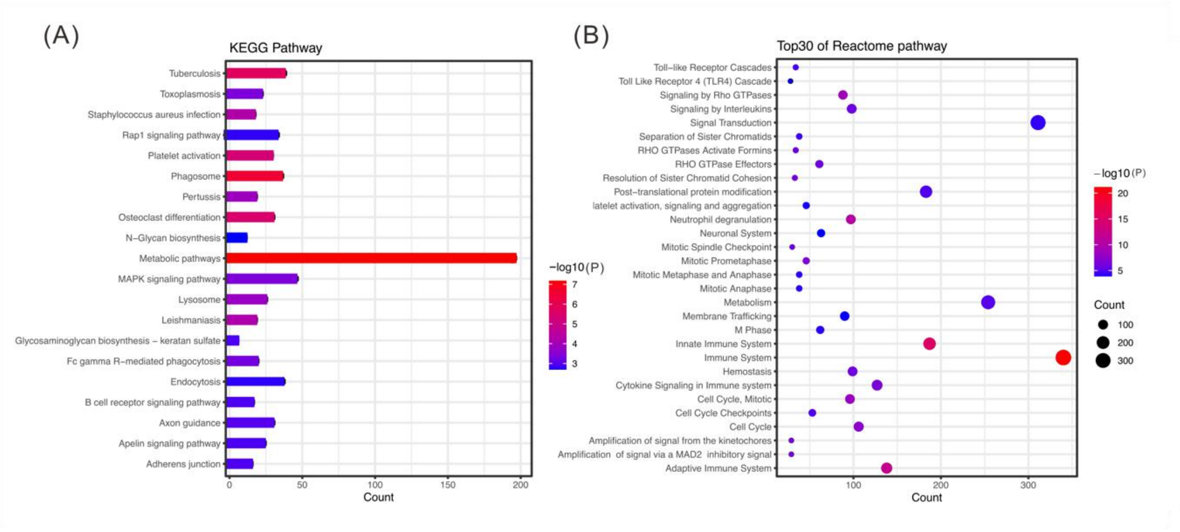

Figure S9. (A) KEGG and (B) Reactome pathway enrichment analysisresult for genes that interacted with two genes in tour signature. The numbers of genes were represented by the length of the bars or the size of the dots, and the color of the bars/dots corresponds to the p-value according to legend.

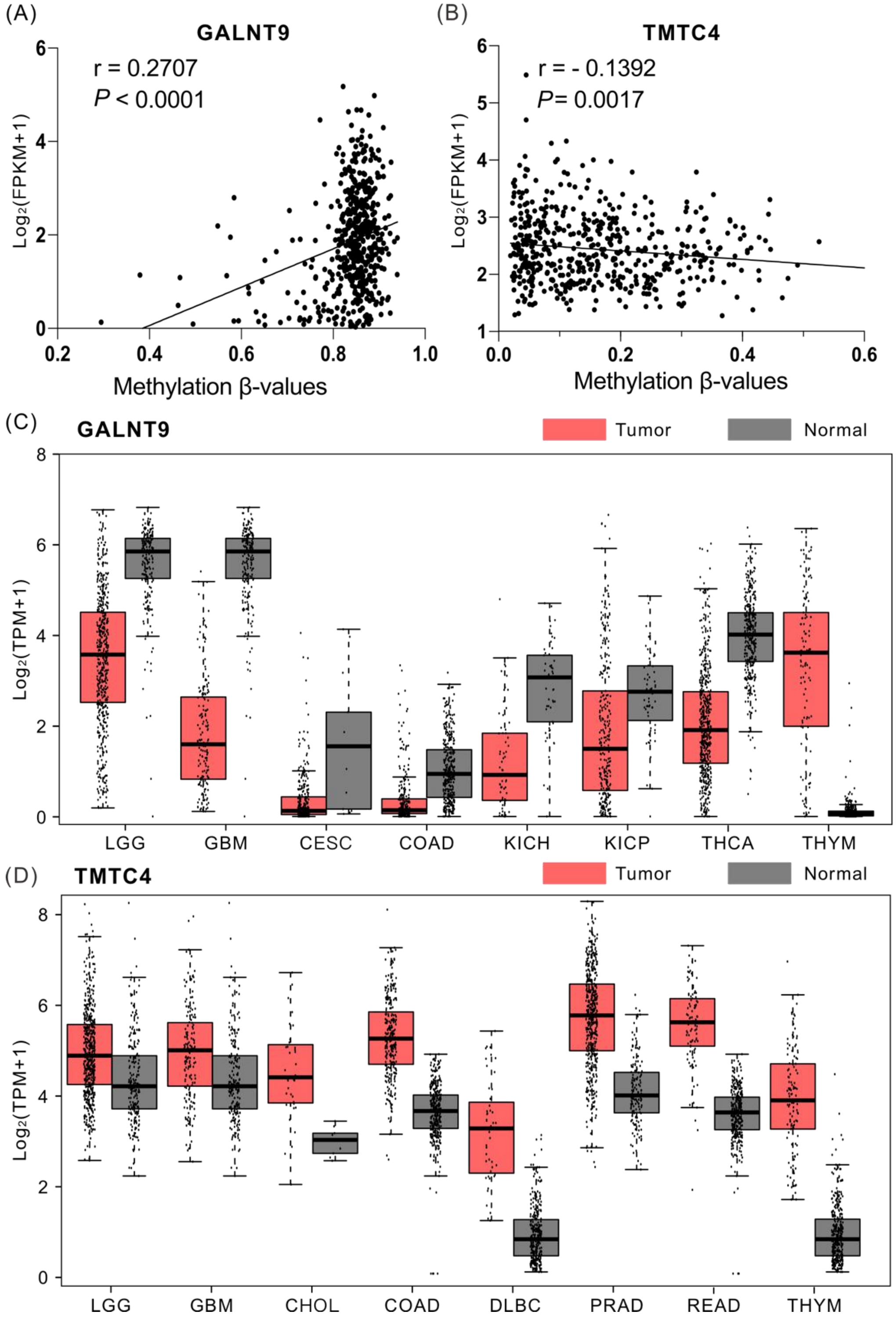

Figure S10. (A-B) Correlation between the expression of the genes and their methylation levels was evaluated for each gene through the Pearson's correlation test. Reported P values are two sided. **(C-D)** The expression of genes in pan-cancer.

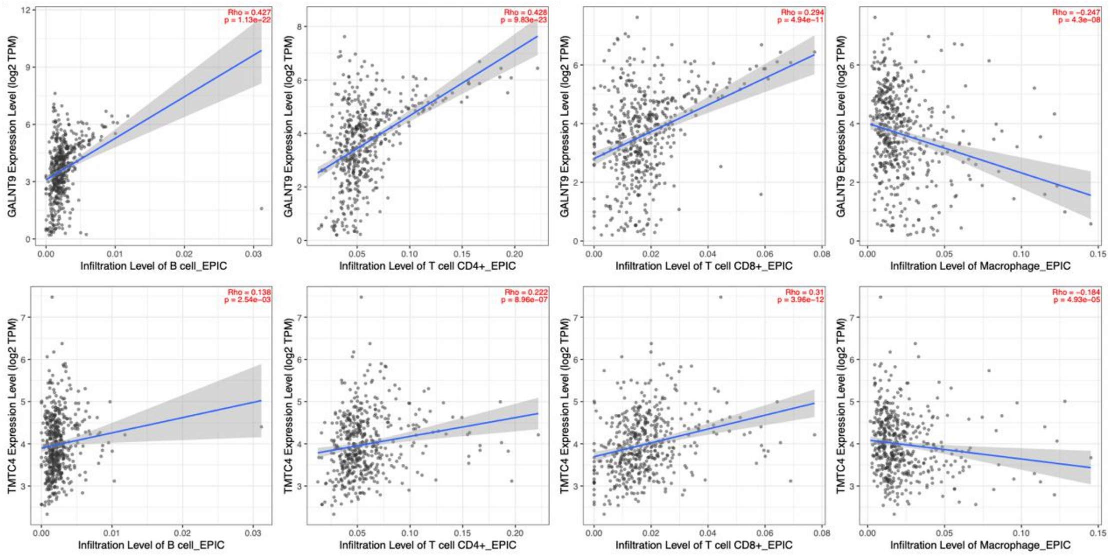

Figure S11. Correlation between the expression of biomarkers from the two-DNA methylation prognostic biomarker and immune cell infiltration level in LGG.

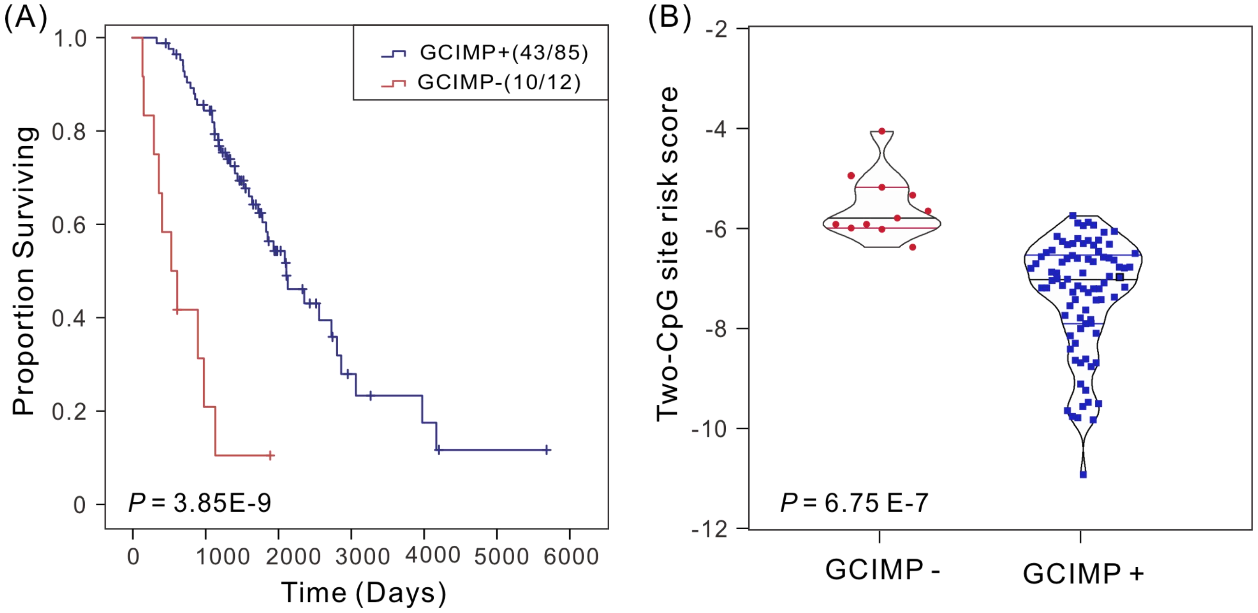

Figure S12. (A) Kaplan–Meier analysis with Log-rank was performed to estimate the differences in OS between the G-CIMP + and G-CIMP- patients. Patients with G-CIMP+ had a favorable prognosis. (B) The violin plot of two-CpG site signature risk scores in G-CIMP + and G-CIMP- patients. Mann-Whitney U test was used to estimate the differences.

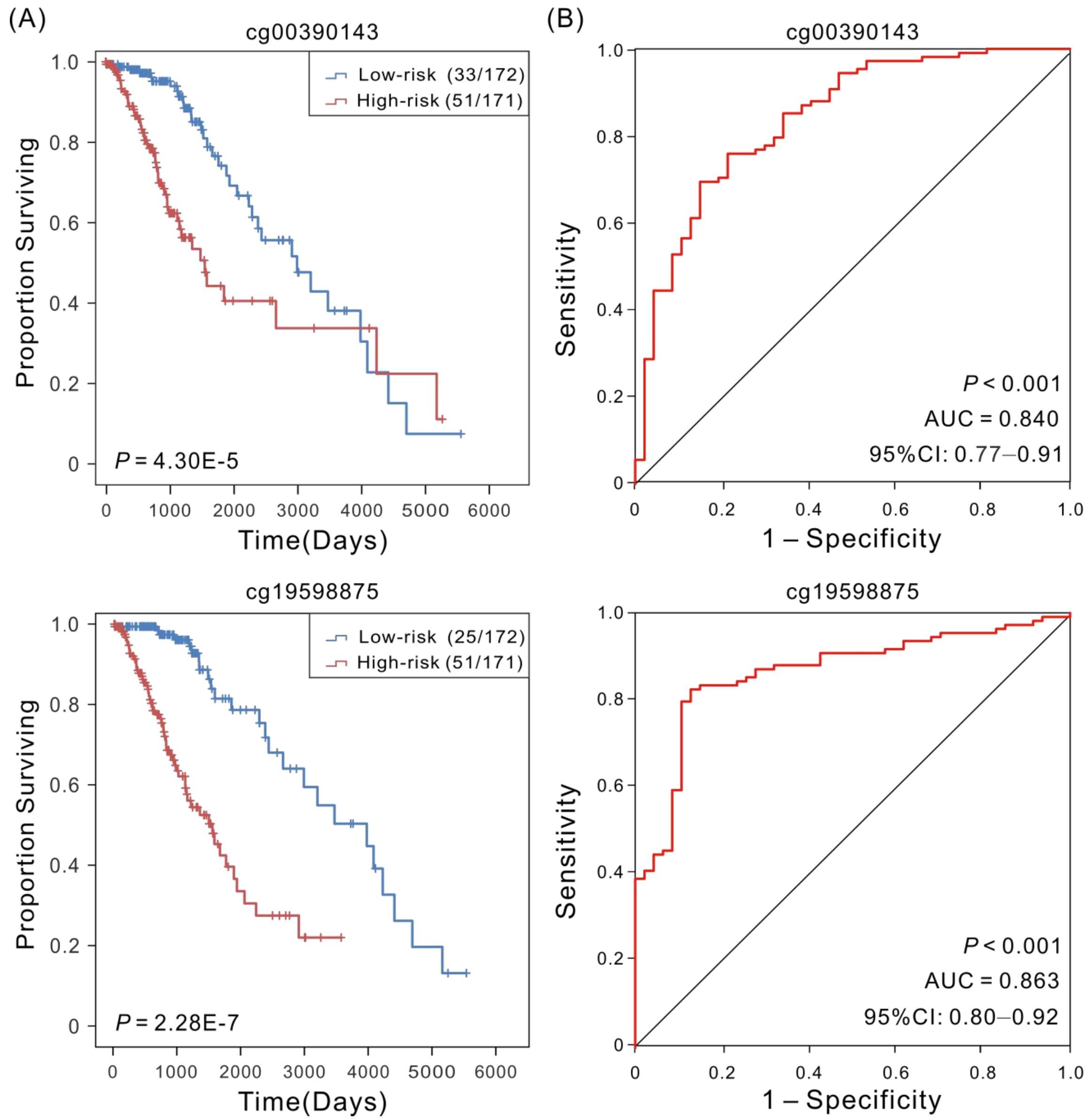

Figure S13. Kaplan–Meier and ROC analyses of individual DNA methylation in the training cohort. (A) Kaplan–Meier analysis with Log-rank was performed to estimate the differences in OS between the low- and high-risk patients. **(B)** ROC curves of the individual methylation signature were used to demonstrate the sensitivity and specificity in predicting the OS of patients.

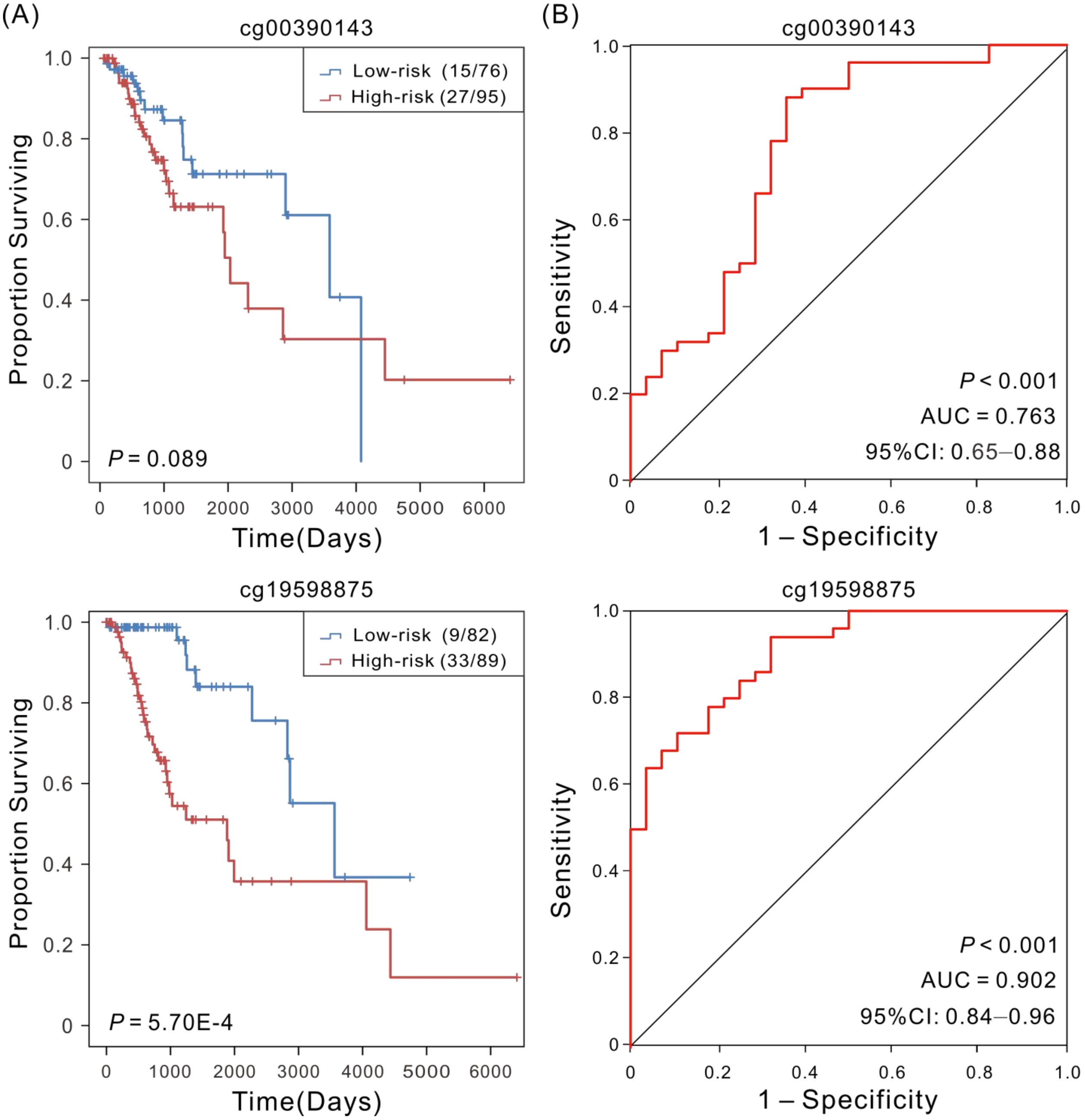

Figure S14. Kaplan–Meier and ROC analyses of individual DNA methylation in the TCGA validation cohort. (A) Kaplan–Meier analysis with Log-rank test was performed to estimate the differences in OS between the low- and high-risk patients. **(B)** ROC curves of the individual methylation signature were used to demonstrate the sensitivity and specificity in predicting the OS of patients.

Additional references

1. Song LR, Weng JC, Huo XL, et al. Identification and validation of a 21-mRNA prognostic signature in diffuse lower-grade gliomas. *J Neurooncol.* Jan 2020;146(1):207-217.
2. Zeng F, Wang K, Liu X, Zhao Z. Comprehensive profiling identifies a novel signature with robust predictive value and reveals the potential drug resistance mechanism in glioma. *Cell Commun Signal.* Jan 6 2020;18(1):2.
3. Zhang M, Wang X, Chen X, Zhang Q, Hong J. Novel Immune-Related Gene Signature for Risk Stratification and Prognosis of Survival in Lower-Grade Glioma. *Front Genet.* 2020;11:363.
4. Deng X, Lin D, Chen B, et al. Development and Validation of an IDH1-Associated Immune Prognostic Signature for Diffuse Lower-Grade Glioma. *Front Oncol.* 2019;9:1310.
5. Zeng WJ, Yang YL, Liu ZZ, et al. Integrative Analysis of DNA Methylation and Gene Expression Identify a Three-Gene Signature for Predicting Prognosis in Lower-Grade Gliomas. *Cell Physiol Biochem.* 2018;47(1):428-439.
6. Binabaj MM, Bahrami A, ShahidSales S, et al. The prognostic value of MGMT promoter methylation in glioblastoma: A meta-analysis of clinical trials. *Journal of cellular physiology.* Jan 2018;233(1):378-386.
7. Rover LK, Gevensleben H, Dietrich J, et al. PD-1 (PDCD1) Promoter Methylation Is a Prognostic Factor in Patients With Diffuse Lower-Grade Gliomas Harboring Isocitrate Dehydrogenase (IDH) Mutations. *EBioMedicine.* Feb 2018;28:97-104.
8. Ma J, Benitez JA, Li J, et al. Inhibition of Nuclear PTEN Tyrosine Phosphorylation Enhances Glioma Radiation Sensitivity through Attenuated DNA Repair. *Cancer cell.* Mar 18 2019;35(3):504-518 e507.
9. Ius T, Ciani Y, Ruaro ME, et al. An NF-kappaB signature predicts low-grade glioma prognosis: a precision medicine approach based on patient-derived stem cells. *Neuro Oncol.* May 18 2018;20(6):776-787.
10. Zhang YA, Zhou Y, Luo X, et al. SHOX2 is a Potent Independent Biomarker to Predict Survival of WHO Grade II-III Diffuse Gliomas. *EBioMedicine.* Nov 2016;13:80-89.
11. Zeng WJ, Yang YL, Wen ZP, Chen P, Chen XP, Gong ZC. Identification of gene expression and DNA methylation of SERPINA5 and TIMP1 as novel prognostic markers in lower-grade gliomas. *PeerJ.* 2020;8:e9262.
12. Vachher M, Arora K, Burman A, Kumar B. NAMPT, GRN, and SERPINE1 signature as predictor of disease progression and survival in gliomas. *J Cell Biochem.* Apr 2020;121(4):3010-3023.
13. Yin AA, Lu N, Etcheverry A, et al. A novel prognostic six-CpG signature in glioblastomas. *CNS Neurosci Ther.* Mar 2018;24(3):167-177.
14. Skiriute D, Steponaitis G, Vaitkiene P, et al. Glioma Malignancy-Dependent NDRG2 Gene Methylation and Downregulation Correlates with Poor Patient Outcome. *Journal of Cancer.* 2014;5(6):446-456.
15. Wiencke JK, Zheng S, Jelluma N, et al. Methylation of the PTEN promoter defines low-grade gliomas and secondary glioblastoma. *Neuro Oncol.* Jul 2007;9(3):271-279.
16. Bady P, Sciuscio D, Diserens AC, et al. MGMT methylation analysis of glioblastoma on the Infinium methylation BeadChip identifies two distinct CpG regions associated with gene silencing and outcome, yielding a prediction model for comparisons across datasets, tumor grades, and CIMP-status. *Acta Neuropathol.* Oct 2012;124(4):547-560.
